# Grey-box modeling framework for consolidated bioprocessing systems: an endpoint-guided approach

**DOI:** 10.1186/s40643-026-01101-9

**Published:** 2026-07-20

**Authors:** Mark Korang Yeboah, Dirk Söffker

**Affiliations:** Chair of Dynamics and Control, University of Duisburg–Essen, Lotharstraße, 47057 Duisburg, NRW Germany

**Keywords:** Consolidated bioprocessing, Grey-box modeling, Endpoint-guided hybrid modeling, Soft sensing, Unscented Kalman filter

## Abstract

**Abstract:**

Consolidated bioprocessing (CBP) combines enzyme production, biomass hydrolysis, and fermentation within a single process, but its modeling remains difficult because of biological nonlinearities, feedstock heterogeneity, and limited time-resolved measurements. This paper presents an endpoint-guided grey-box framework that connects data-driven endpoint prediction, phase-structured mechanistic reconstruction, and synthetic state-estimation analysis. A literature-derived CBP ethanol dataset containing 540 runs and 90 encoded input features was preprocessed using out-of-fold residual screening, after which several nonlinear regressors were compared using both log-transformed and raw endpoint targets. Repeated cross-validation selected a raw-target XGBoost model, XGB_raw, as the final endpoint surrogate, with RMSE *=1.566 ± 0.465*. The independent hold-out subset favored histogram-based gradient boosting models, indicating that the leading boosting-based models were closely matched. For XGB_raw, hold-out performance improved after residual screening from RMSE *=7.491*, MAE *=2.798*, and $R^2=0.821$ to RMSE *=2.025*, MAE *=1.253*, and $R^2=0.940$. Feature-attribution analysis identified hemicellulose content, substrate concentration, temperature, residence time, enzyme-assisted pretreatment, pH, cellulose content, and mixing rate as important endpoint predictors. The selected endpoint surrogate was then coupled to a three-phase hybrid simulator representing enzyme production, hydrolysis, and fermentation. Endpoint-guided calibration identified biologically plausible parameterizations that reproduced the target endpoint through moderate changes in growth, enzyme production, hydrolytic capacity, and product formation. Because independent time-resolved CBP trajectories were unavailable, the simulated profiles are interpreted as endpoint-constrained reconstructions rather than validated kinetic trajectories. A synthetic unscented Kalman filter study showed accurate reconstruction of sugar and scaled-product states, with lower-fidelity recovery of enzyme dynamics. Overall, the framework provides a feasibility-oriented basis for CBP endpoint prediction, mechanistic interpretation, and preliminary soft-sensing design under sparse-data conditions.

**Graphical Abstract:**

**Figure:**
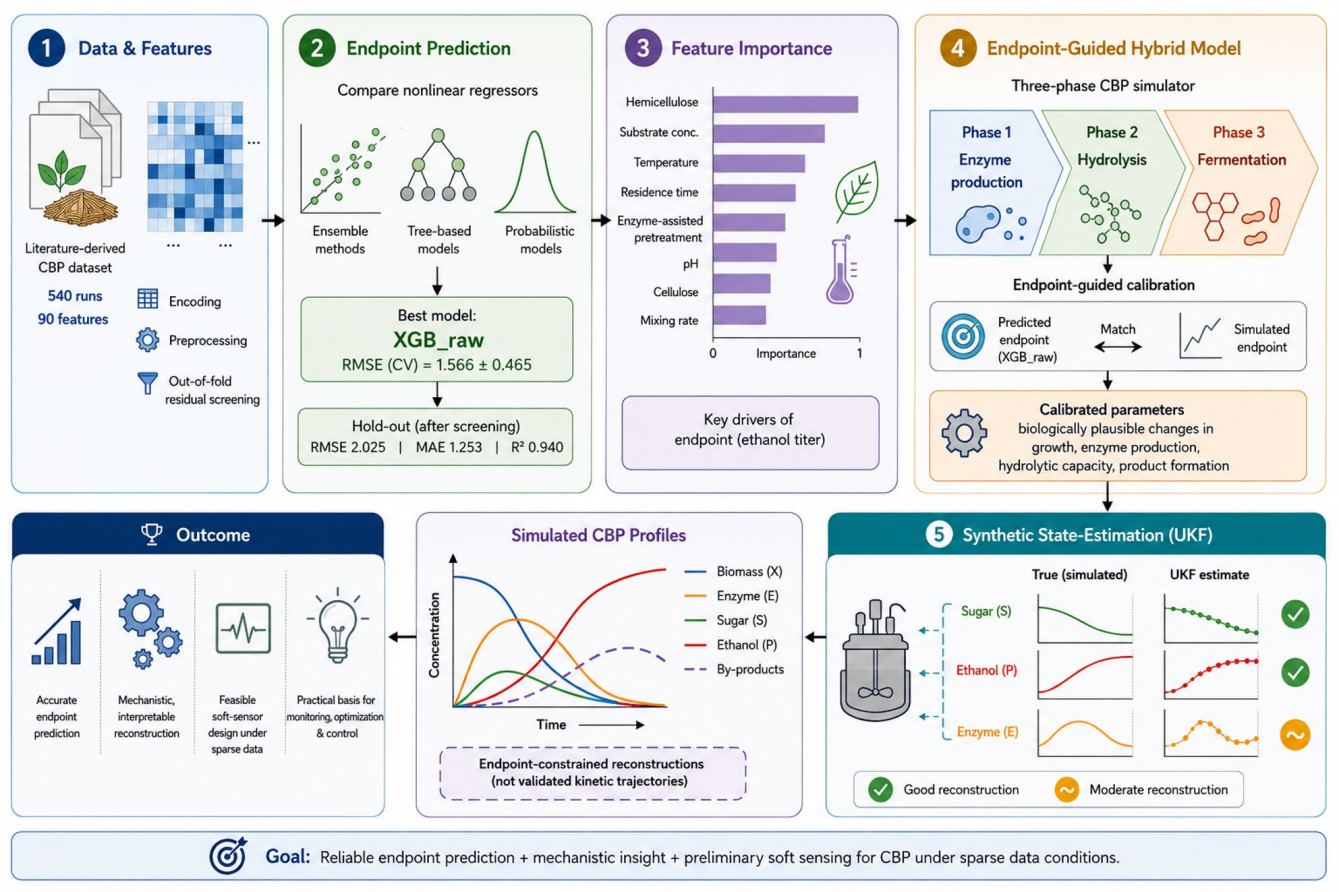

**Electronic supplementary material:**

The online version of this article (10.1186/s40643-026-01101-9) contains supplementary material, which is available to authorized users.

## Introduction

Consolidated bioprocessing (CBP) combines enzyme production, biomass hydrolysis, and fermentation within a single process and has been widely recognized as a promising route for converting lignocellulosic biomass into fuels and biochemicals (Yeboah and Söffker 2026; Sharma et al. 2022). Its practical implementation, however, remains challenging because process performance is jointly governed by microbial activity, substrate accessibility, feedstock composition, and operating conditions such as temperature and pH (Wen et al. 2020; Cheng et al. 2023). These coupled effects make CBP highly nonlinear and difficult to analyze, optimize, and monitor within a consistent modeling framework.

Mathematical models are essential for interpreting bioprocess behavior and supporting future optimization, monitoring, and control strategies. Mechanistic models are physically interpretable, but they are difficult to calibrate reliably for CBP because many interacting biological effects are only partly understood and experimental observations are often limited (Pérez et al. 2020; Rathore et al. 2021). Purely data-driven models can capture nonlinear input–output behavior, but they typically require larger and more homogeneous datasets than are available for CBP, and their limited transparency reduces their value for diagnosis and process understanding (Cheng et al. 2023; Yeboah et al. 2024). In studies based on literature-derived CBP data, this difficulty is further amplified because results are often reported as final titers or yields rather than as densely sampled dynamic trajectories.

Grey-box and hybrid modeling provide a useful compromise between fully mechanistic and purely data-driven approaches. By preserving first-principles structure while allowing data-driven components to represent unresolved nonlinearities, hybrid models can remain interpretable without becoming overly rigid (Pérez et al. 2020; Agharafeie et al. 2023). This balance has been valuable in broader bioprocess and biochemical engineering applications, particularly when process knowledge is incomplete and measurements are sparse (von Stosch et al. 2014; Narayanan et al. 2022). The same need is evident in CBP, where enzyme production, hydrolysis, and fermentation are tightly coupled, but published studies generally report endpoint quantities such as final titer or yield rather than detailed dynamic trajectories.

Recent machine-learning studies in bioprocess engineering have shown the value of comparing multiple nonlinear predictors rather than relying on a single surrogate family (Duong-Trung et al. 2023; Mondal et al. 2023; Jurinjak Tušek et al. 2024). Endpoint-prediction benchmarks are especially relevant for ensemble methods and Gaussian-process models because these models can capture nonlinear effects of operating conditions and feedstock descriptors. However, endpoint prediction alone does not reconstruct the underlying CBP trajectory. At the same time, soft-sensor and state-estimation studies have highlighted the importance of estimating internal variables that are difficult to measure directly in biochemical and biosystem monitoring (Alexander et al. 2020; Lyubenova et al. 2021; Badreldin et al. 2024). These developments motivate a workflow that links endpoint prediction, dynamic reconstruction, and preliminary state-estimation feasibility within a single CBP modeling framework.

Recent data-driven CBP studies have also extended literature-derived endpoint modeling beyond ethanol alone. For example, Yeboah et al. (2026a) developed a multi-product CBP learning framework for ethanol and co-products, showing that product learnability depends strongly on target support, missing-label structure, and cross-study heterogeneity. However, such endpoint-level frameworks do not directly reconstruct mechanistic CBP trajectories. In parallel, recent CBP digital-twin work has shown that measurement-set selection can strongly affect state observability, parameter identifiability, and unscented Kalman filter (UKF)-based soft-sensing performance, highlighting the importance of sensor availability for future experimental deployment (Yeboah et al. 2026c). These findings further support the need for a framework that connects endpoint learning with interpretable dynamic reconstruction and state-estimation feasibility.

Against this background, the relevant literature reveals a clear methodological gap. Mechanistic models support biological interpretation but are difficult to calibrate under limited observability, while data-driven models can describe nonlinear endpoint behavior without directly recovering plausible internal dynamics (Yeboah and Söffker 2026; Cheng et al. 2023). More broadly, recent hybrid-modeling studies in bioprocess engineering demonstrate the benefit of combining mechanistic and statistical descriptions, but such frameworks have rarely been adapted to heterogeneous, literature-derived CBP datasets (Agharafeie et al. 2023; Narayanan et al. 2022). A remaining need is a workflow that uses sparse endpoint data not only for prediction but also to guide mechanistically interpretable dynamic reconstruction and preliminary soft-sensing analysis within a coherent CBP framework.

In the present work, an endpoint-guided grey-box framework is developed for literature-derived CBP data. Final ethanol prediction is formulated as a data-driven endpoint-learning problem, in which several nonlinear regressors are trained and compared to provide an internal benchmark across common machine-learning surrogate families and target transformations. The final endpoint surrogate is selected using repeated cross-validation on the training subset, while the hold-out subset is reserved for independent performance reporting. The selected endpoint predictor is then coupled externally to a phase-structured mechanistic simulator describing enzyme production, biomass hydrolysis, and fermentation. This coupling enables endpoint-guided calibration of biologically plausible dynamic reconstructions rather than full kinetic validation from time-resolved experimental data. Finally, the calibrated hybrid structure is evaluated in a synthetic unscented Kalman filter feasibility study to examine its potential for future soft-sensing applications. Thus, the novelty of the present study lies in linking repeated-cross-validation-based endpoint prediction, endpoint-constrained mechanistic reconstruction, and preliminary synthetic state-estimation analysis within a single workflow for data-limited CBP systems.

## Methodology

### Background of consolidated bioprocessing system

Consolidated bioprocessing combines three biochemical steps into a single reactor: cellulolytic enzyme production, biomass hydrolysis, and fermentation. This integration reduces process complexity and limits the requirement for externally added enzymes compared with traditional multi-stage routes (Cheng et al. 2023; Sharma et al. 2022). This configuration is especially relevant for lignocellulosic biomass conversion, where feedstock recalcitrance, operational costs, and process integration are still major challenges (Liu et al. 2020; Singhania et al. 2022). In CBP, a microbial strain or consortium should produce hydrolytic enzymes and convert the released sugars into target products such as ethanol or organic acids. Thus, process performance depends on biological capability and operating conditions such as temperature, pH, substrate loading, and pretreatment quality (Singhania et al. 2022; Yeboah et al. 2024).

From a modeling standpoint, CBP is difficult because enzyme production, hydrolysis, and fermentation happen simultaneously and influence each other via strongly nonlinear mechanisms. These interactions include sugar release kinetics, substrate inhibition, product feedback, enzyme deactivation, and phase-dependent microbial activity, all of which are difficult to model reliably with a purely mechanistic model, especially with limited observability (Minnaar and den Haan 2023; Li et al. 2024). The reactor configuration, mixing, and pretreatment of the feedstock also impact the accessibility of the substrate and the overall efficiency of the conversion (Yeboah and Söffker 2026; Liu et al. 2020). Thus, CBP is well-suited for grey-box modeling, where a phase-structured mechanistic model maintains known process relationships, while a data-driven endpoint predictor guides parameter selection in the presence of unresolved nonlinearities, variability, and limited observability.

This modeling choice is in line with general trends in hybrid bioprocess modeling studies, which combine mechanistic structures with statistical or machine-learning components to improve prediction while preserving interpretability (von Stosch et al. 2014; Narayanan et al. 2022). The present work does not intend to replace organism-specific kinetic identification with the grey-box simulator. Rather, it offers a dynamic, control-oriented, and interpretation-oriented scaffold, constrained by endpoint information from heterogeneous CBP studies.

### Model structure and phase-structured architecture for CBP systems

The dynamic complexity of CBP is due to three biologically distinct but temporally overlapping regimes, namely enzyme production, biomass hydrolysis, and microbial fermentation, occurring within a single reactor. Coupled CBP dynamics are represented using a mechanistic formulation with phase-specific kinetic components combined through smooth activation functions, while remaining interpretable. The dynamic simulator is coupled to a separate data-driven endpoint predictor, which guides parameter selection at the process endpoint. Thus, the overall CBP model is expressed as a weighted sum of three dynamic components, each corresponding to a phase. Smooth phase-activation functions guarantee a gradual transition between regimes and prevent discontinuities.

The architecture incorporates an internal bridge between endpoint learning and dynamic reconstruction. The endpoint model identifies regions of operating conditions associated with high final ethanol production, and the phase-structured simulator translates endpoint-consistent parameter choices into interpretable trajectories of biomass, enzyme, insoluble substrate, soluble sugar, and product. This separation is useful for CBP data derived from the literature because endpoint values are more readily available than dense time-series measurements.

#### Overall hybrid model

Let $\textbf{x}(t)$ denote the state vector and let $\boldsymbol{\theta }_i$ denote the parameter set associated with phase *i*. The dynamic model is written as a weighted combination of three phase-specific components as

1 $$\begin{aligned} \frac{d\textbf{x}(t)}{dt} = \sum _{i=1}^{3} \phi _i(t)\,f_i\!\bigl (\textbf{x}(t), \boldsymbol{\theta }_i\bigr ), \end{aligned}$$

where the indices $i \in \{1,2,3\}$ correspond to enzyme production, hydrolysis, and fermentation, respectively, and $f_i(\cdot )$ denotes the phase-specific dynamic contribution. The activation functions $\phi _i(t)$ satisfy $\phi _i \in [0,1]$ and $\sum _{i=1}^{3}\phi _i = 1$, thereby enabling smooth transitions between regimes over time.

The state vector is defined as

2 $$\begin{aligned} \textbf{x}(t) = \begin{bmatrix} X&E&B&C&P \end{bmatrix}^{\textsf{T}}, \end{aligned}$$

where *X*, *E*, *B*, *C*, and *P* denote microbial biomass concentration (g/L), enzyme concentration (U/mL), insoluble substrate concentration (g/L), soluble sugar concentration (g/L), expressed here as an effective glucose-equivalent lumped state, and bioproduct concentration (g/L), respectively.

The selected state representation is also consistent with recent reduced-order dynamic CBP optimization work, in which biomass, enzyme activity, insoluble substrate, soluble sugar, and ethanol were used as compact state variables for model-based evaluation of batch CBP operation under varying temperature and pH policies (Yeboah et al. 2026b). However, the role of the dynamic model in the present study is different. Whereas Yeboah et al. (2026b) used the reduced-order model for feasibility-aware Pareto optimization of operating policies, the present work uses a phase-structured grey-box simulator as an endpoint-guided reconstruction layer. Thus, the simulator is not used here to identify optimal temperature–pH schedules, but rather to generate biologically interpretable trajectories that are consistent with endpoint behavior learned from heterogeneous literature-derived CBP data.

Role of the endpoint predictor In this workflow, the endpoint predictor is not embedded directly in Eq. (1). Instead, Eq. (1) defines the phase-structured mechanistic core of the grey-box framework, while the data-driven endpoint model acts as an external calibration signal for selecting parameter realizations that reproduce endpoint behavior learned from the literature-derived dataset. This separation preserves the interpretability of the dynamic equations and allows endpoint data to guide calibration when full trajectory measurements are not available.

#### Model 1: enzyme production phase

In the CBP enzyme-production regime, the biomass (microorganisms) grows and secretes the hydrolytic enzymes needed for the later depolymerization of biomass. Biomass growth is governed by a logistic-type law with first-order endogenous decay. A biomass-associated production term and an explicit first-order inactivation term govern enzyme accumulation. This formulation separates enzyme production from enzyme loss, avoiding the confounding of secretion with deactivation as

3 $$\begin{aligned} \frac{dX}{dt}&= \Big [\mu _1(T, pH, \textbf{u}) - \mu _{d,1}\Big ]\,X\left( 1 - \frac{X}{K}\right) \end{aligned}$$

and

4 $$\begin{aligned} \frac{dE}{dt}&= q_E(T, pH, \textbf{u})\,X - k_{\deg }(T, pH, \textbf{u})\,E, \end{aligned}$$

where *X*, *E*, *T*, and *pH* denote microbial biomass concentration (g/L), enzyme concentration (U/mL), temperature ($^\circ$C), and reactor pH, respectively. The effective specific growth-rate coefficient $\mu _1(\cdot )$ has units of 1/h, $\mu _{d,1}$ denotes the endogenous decay rate (1/h), and *K* is the biomass carrying capacity (g/L), which acts as the upper bound in the logistic growth term and limits nonphysical biomass escalation during the enzyme-production phase. The function $q_E(\cdot )$ denotes the specific enzyme production rate with units of U g$^{-1}$ h$^{-1}$. The enzyme inactivation rate $k_{\deg }(\cdot )$ (1/h) includes thermal and pH effects and other losses depending on operating conditions. This formulation follows standard bioprocess kinetic descriptions of logistic microbial growth, biomass-associated product formation, and lumped enzyme loss (Tsoularis and Wallace 2002; Luedeking and Piret 1959; Bailey and Ollis 1986).

Additional process descriptors are grouped in *u*, including substrate composition, pretreatment information, inoculum-related descriptors, and mixing conditions, where available. Oxygen is not represented as a separate state variable in the present formulation; its possible influence is instead absorbed into the lumped kinetic terms and the available process descriptors. The enzyme-production phase is therefore treated as a compact, interpretable biological submodel that captures the dependence of downstream hydrolysis capacity on upstream biomass growth and enzyme accumulation, without requiring detailed intracellular or regulatory descriptions that are rarely available in literature-derived CBP datasets. In the present implementation, the growth, enzyme-production, and enzyme-loss terms are not identified directly from dynamic measurements. Their effective values are conditioned through the available operating descriptors and then adjusted through the endpoint-guided multiplier search.

#### Model 2: biomass hydrolysis phase

Secreted enzymes depolymerize insoluble lignocellulosic substrate into soluble sugars during the hydrolysis regime. Hydrolysis is represented with a lumped enzyme-mediated rate expression that captures this stage in a compact mechanistic description by linking soluble-sugar release to substrate depletion through an effective stoichiometric yield. This formulation captures the dominant coupling among enzyme concentration, substrate availability, and sugar release while retaining a physically interpretable mass balance as

5 $$\begin{aligned} \frac{dC}{dt}&= r_{\text {hyd}}(B,E;T,pH) \end{aligned}$$

and

6 $$\begin{aligned} \frac{dB}{dt}&= -\frac{1}{Y_{C/B}}\,r_{\text {hyd}}(B,E;T,pH), \end{aligned}$$

where *C*, *B*, and $Y_{C/B}$ denote soluble sugar concentration in $\mathrm {g_{sugar}\,L^{-1}}$, expressed here as an effective glucose-equivalent lumped state, insoluble substrate concentration in $\mathrm {g_{substrate}\,L^{-1}}$, and the effective soluble-sugar release yield in $\mathrm {g_{sugar}\,g_{substrate}^{-1}}$, respectively. Therefore, $r_{\text {hyd}}$ represents the soluble-sugar formation rate in $\mathrm {g_{sugar}\,L^{-1}\,h^{-1}}$, while *dB*/*dt* represents the corresponding insoluble-substrate depletion rate in $\mathrm {g_{substrate}\,L^{-1}\,h^{-1}}$.

A convenient lumped mechanistic representation is given by

7 $$\begin{aligned} r_{\text {hyd}}(B,E;T,pH) = V_{\max }(T,pH)\,E\,\frac{B}{K_m + B}, \end{aligned}$$

where *E* denotes the enzyme activity concentration in $\mathrm {U\,mL^{-1}}$, $K_m$ the apparent substrate-saturation constant in $\mathrm {g_{substrate}\,L^{-1}}$, and $V_{\max }(T,pH)$ an apparent maximum hydrolysis coefficient normalized by enzyme activity, with units of $\mathrm {g_{sugar}\,L^{-1}\,h^{-1}\,(U\,mL^{-1})^{-1}}$. Accordingly, the product $V_{\max }E$ has units of $\mathrm {g_{sugar}\,L^{-1}\,h^{-1}}$, whereas the ratio $B/(K_m+B)$ is dimensionless because both *B* and $K_m$ are expressed in $\mathrm {g_{substrate}\,L^{-1}}$. Therefore, Eq. (7) is dimensionally consistent with Eq. (5), while Eq. (6) converts the soluble-sugar formation flux into the corresponding insoluble-substrate depletion flux through $Y_{C/B}$.

In the current study, the Michaelis–Menten-type formulation is adopted as a lumped approximation of enzyme-mediated hydrolysis. This form preserves the expected dependence on both enzyme availability and accessible substrate, while remaining identifiable under sparse endpoint-guided calibration. The yield term $Y_{C/B}$ represents the effective conversion from insoluble-substrate depletion to soluble-sugar release and does not imply a direct one-to-one equivalence between the two rates. In this formulation, $r_{\text {hyd}}(\cdot )$ denotes the net soluble-sugar release flux, such that soluble-sugar formation and insoluble-substrate depletion remain mutually consistent through Eq. (6). The lumped Michaelis–Menten-type hydrolysis expression follows standard biochemical-engineering descriptions of enzyme-mediated substrate conversion and is consistent with common kinetic treatments of enzymatic cellulose and lignocellulosic-biomass hydrolysis (Bailey and Ollis 1986; Zhang and Lynd 2004; Jeoh et al. 2017).

#### Model 3: fermentation phase

Microorganisms use soluble sugars in fermentation to support growth and production of desired bioproducts, such as ethanol or organic acids. The current formulation describes biomass growth with a substrate-limited and product-inhibited specific growth rate, biomass loss with a first-order decay term, and product formation as a growth-associated term with an additional first-order consumption or degradation term. Biomass growth and product formation are both dependent on sugar consumption through yield coefficients as

8 $$\begin{aligned} \frac{dX}{dt}&= \mu _3(C,P,T,pH,\textbf{u})\,X - \mu _{d,3}\,X, \end{aligned}$$

9 $$ \begin{aligned} \frac{{dP}}{{dt}} = & Y_{{P/X}} (T,pH,{\mathbf{u}}){\mkern 1mu} \mu _{3} (C,P,T,pH,{\mathbf{u}}){\mkern 1mu} X \\ & - k_{{P,{\mathrm{cons}}}} (T,pH,{\mathbf{u}}){\mkern 1mu} P \\ \end{aligned} $$

and

10 $$ \begin{aligned} \frac{{dC}}{{dt}} = & - \frac{1}{{Y_{{X/C}} }}{\mkern 1mu} \mu _{3} (C,P,T,pH,{\mathbf{u}}){\mkern 1mu} X \\ & - \frac{1}{{Y_{{P/C}} }}{\mkern 1mu} Y_{{P/X}} (T,pH,{\mathbf{u}}){\mkern 1mu} \mu _{3} (C,P,T,pH,{\mathbf{u}}){\mkern 1mu} X, \\ \end{aligned} $$

where $\mu _3(\cdot )$, $\mu _{d,3}$, $Y_{P/X}(\cdot )$, and $k_{P,\text {cons}}(\cdot )$ denote the fermentation-specific growth rate (1/h), endogenous decay rate (1/h), growth-associated product yield (g product/g biomass formed), and product consumption or degradation rate (1/h), respectively. In Eq. (10), the yields $Y_{X/C}$ (g biomass/g sugar) and $Y_{P/C}$ (g product/g sugar) couple biomass growth and product formation to soluble-sugar uptake. This representation provides a compact description of the downstream conversion stage and yet remains mechanistically interpretable in terms of substrate utilization, biomass formation, and product accumulation. This fermentation submodel follows standard unstructured fermentation kinetics, where microbial growth is substrate-limited, product accumulation is linked to growth-associated formation, and product inhibition or loss is represented through lumped kinetic terms (Bailey and Ollis 1986; Luedeking and Piret 1959; Starzak et al. 1994).

#### Phase activation functions

The three hybrid submodels describe different biological regimes in CBP, i.e., enzyme production, hydrolysis, and fermentation, but are combined into a single continuous framework by means of smooth phase-weighting functions. The integration is necessary because CBP is conducted as a coupled continuum, where enzyme production provides catalytic capacity, hydrolysis produces fermentable sugar, and fermentation consumes sugar and produces a target metabolite. The smooth weighting avoids discontinuous switching, improves numerical stability, and allows for temporal overlap between regimes (Mowbray et al. 2023). It also allows a more realistic representation of CBP behavior, because these biological stages are not sharply separated in practice but rather overlap over the course of the batch.

The use of smooth phase-dependent dynamics is motivated by the fact that CBP does not proceed through sharply separated biological stages. Enzyme formation, substrate hydrolysis, soluble-sugar accumulation, fermentation, and product inhibition can overlap during batch operation. A related CBP optimization study represented these coupled processes using a reduced-order dynamic model and showed that temperature and pH affect growth, hydrolysis, and fermentation in conflicting ways (Yeboah et al. 2026b). The present formulation follows the same general interpretation of CBP as a coupled dynamic system, but introduces endpoint-guided calibration to make the model usable when dense time-resolved measurements are unavailable.

**Fig. 1:**
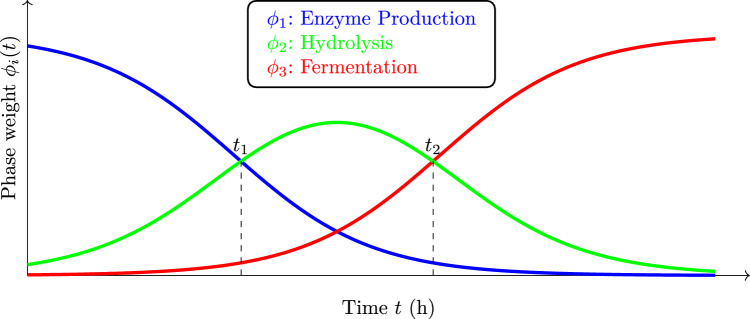
Phase-weighting functions for the hybrid CBP model. Sigmoid curves $\phi _1$, $\phi _2$, and $\phi _3$ represent the relative influence of enzyme production, hydrolysis, and fermentation over time. Dashed lines $t_1$ and $t_2$ indicate the intersection-based phase transitions

In the present implementation, phase weights depend on time and are defined using sigmoid functions as

11 $$\begin{aligned} \phi _1(t)&= \frac{1}{1+\exp \!\bigl (k\,(t-t_1)\bigr )}, \end{aligned}$$

12 $$\begin{aligned} \phi _3(t)&= \frac{1}{1+\exp \!\bigl (-k\,(t-t_2)\bigr )} \end{aligned}$$

and

13 $$ \begin{aligned} \phi _{2} (t) = & 1 - \phi _{1} (t) - \phi _{3} (t)\; = \;\frac{1}{{1 + \exp ( - k{\mkern 1mu} (t - t_{1} ))}}\; \\ & - \;\frac{1}{{1 + \exp ( - k{\mkern 1mu} (t - t_{2} ))}}, \\ \end{aligned} $$

where $t_1$ and $t_2$ denote the centers of the transitions from enzyme production to hydrolysis and from hydrolysis to fermentation, respectively, as shown in Fig. 1, and *k>0* controls the transition steepness. For $t_2>t_1$, the construction ensures that $\phi _i(t)\in [0,1]$ and $\sum _{i=1}^{3}\phi _i(t)=1$ for all *t*, with $\phi _2(t)\ge 0$ acting as a smooth intermediate “window” between the two transitions. State-dependent phase activation, $\phi _i(t,\textbf{y})$, can be incorporated analogously when transition timing is governed by process conditions rather than time alone. The use of smooth sigmoid-type switching functions is consistent with hybrid dynamical-system and biological-process modeling, where gradual activation is often used to avoid discontinuous switching while preserving interpretable regime changes (Ghosh and Tomlin 2004; Bortolussi and Policriti 2008; Mahanty 2023).

### Dataset and experimental variables

The endpoint dataset comprised 540 literature-derived CBP ethanol runs, summarized by biomass/reference group in Table S1 of the Supplementary Materials. Only records with a reported final ethanol titer and sufficient process information for feature encoding were retained. Duplicate records, non-CBP experiments, and records with insufficient documentation were excluded. The resulting dataset spans temperatures of *25*–$75~{}^\circ \textrm{C}$, pH values of *3.0*–*9.0*, residence times of *6*–$3504~\textrm{h}$, substrate concentrations of *2.0*–$366.7~\mathrm {g\,L^{-1}}$, working volumes of *0.004*–$4.0~\textrm{L}$, and mixing rates of *0*–$400~\textrm{rpm}$, where reported. It includes lignocellulosic, model-carbohydrate, and other feedstocks, multiple pretreatment classes, and both monoculture and co-culture systems. The full encoded feature catalogue is provided in Table S2.

Each row represents one CBP run; therefore, all predictors are treated as run-level descriptors rather than time-resolved measurements. The final matrix contains 90 input features and one output variable, the endpoint ethanol titer. Predictors include temperature, pH, pretreatment status, substrate loading, residence time, mixing variables, inoculum- and volume-related descriptors, compositional variables where available, and one-hot-encoded categorical descriptors. Numeric fields were standardized to a common format, Boolean variables were encoded as integers, remaining categorical variables were one-hot encoded, infinite values were removed, and missing values were imputed using feature-wise medians. This procedure produced a unified tabular representation suitable for cross-sectional endpoint prediction (Fisher et al. 2022; Schweidtmann et al. 2024; Duong-Trung et al. 2023; Jurinjak Tušek et al. 2024).

The full encoded feature set was used for endpoint learning, whereas a smaller mechanistically interpretable subset comprising temperature, pH, pretreatment status, and substrate concentration was used to condition the grey-box simulator. The nominal phase-specific coefficients and environmental modifiers used in the hybrid model are summarized in Table 1. This separation preserves the distinction between endpoint prediction and dynamic reconstruction when dense time-resolved measurements are unavailable (Wang et al. 2023; Agharafeie et al. 2023; von Stosch et al. 2014).

**Table 1:** Selected nominal phase-specific baseline coefficients and environmental modifier functions used in the hybrid model

Phase	Parameter	Baseline	Conditioned form
Enzyme production	$\mu _{1,\max }$	0.20	$0.20\,f_T(T)\,f_{\textrm{pH}}(\textrm{pH})$
	$q_{E,\max }$	0.06	$0.06\,f_T(T)\,f_{\textrm{pH}}(\textrm{pH})\,f_{\textrm{pret}}(\textrm{pret})$
Hydrolysis	$V_{\max }^{*}$	0.20	$0.20\,f_T(T)\,f_{\textrm{pH}}(\textrm{pH})\,f_{\textrm{conc}}(\textrm{conc})$
	$K_m^{*}$	0.8	$0.8\,f_{\textrm{pret}}(\textrm{pret})$
Fermentation	$\mu _{3,\max }$	0.12	$0.12\,f_T(T)\,f_{\textrm{pH}}(\textrm{pH})$
	$Y_{P/X}$	0.30	$0.30\,f_T(T)\,f_{\textrm{pH}}(\textrm{pH})$

Before model training, iterative out-of-fold (OOF) residual screening was used as a data-curation step to reduce the influence of high-disagreement endpoint records in the heterogeneous literature-derived corpus. Removed and retained samples were compared using supplementary feature-distribution diagnostics, and the samples removed between the 10 % and 15 % screening levels were analyzed separately because this interval produced a nonmonotonic increase in OOF RMSE. These diagnostics are reported in the Supplementary Materials and are used to distinguish possible anomalous records from valid but underrepresented regions of the CBP feature space.

The retained subset was divided using an 80/20 split. The training portion was used for cross-validated model comparison and fitting, while the hold-out subset was reserved for independent performance reporting and residual diagnostics. No external endpoint-validation dataset beyond the compiled 540-run literature corpus was available; therefore, the hold-out subset provides an internal generalization test, while external endpoint validation and time-resolved dynamic validation remain future requirements.

#### Exploratory Spearman ranking of endpoint-learning features

Spearman’s rank correlation was used as a model-independent exploratory ranking of the monotonic association between each encoded predictor and endpoint ethanol titer (Spearman 1904). Features were ranked by absolute correlation, *|ρ |*, and *95~%* bootstrap confidence intervals were computed by repeated resampling. This analysis was used only to summarize dominant univariate trends in the dataset. It was not used as a feature-selection rule because rank correlation cannot capture multivariable interactions, nonlinear thresholds, or feature dependencies learned by the nonlinear endpoint models (Duong-Trung et al. 2023; Jurinjak Tušek et al. 2024).

### Parameter estimation and model training

**Fig. 2:**
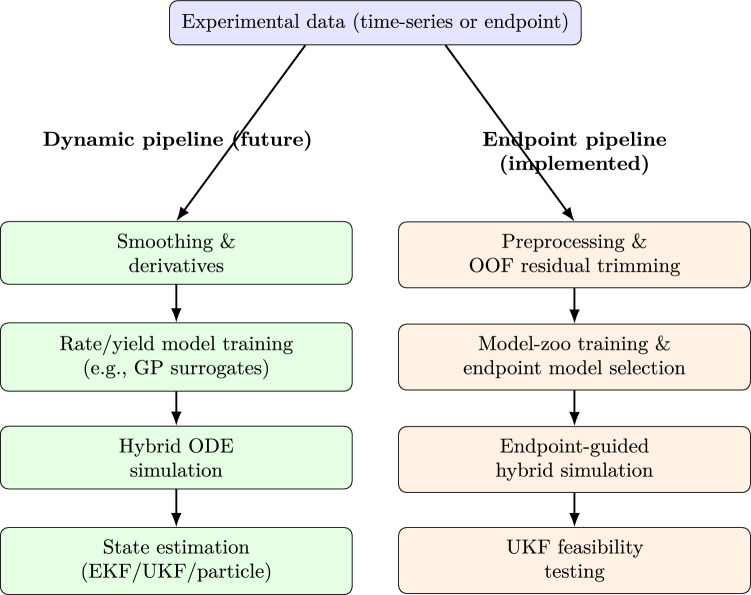
Two complementary parameter-estimation pipelines. Left: conceptual dynamic pipeline using time-series data to estimate phase-dependent rates, simulate the hybrid ODE system, and perform recursive state estimation. Right: implemented endpoint pipeline using cross-sectional data preprocessing, out-of-fold residual trimming, model-zoo endpoint training and selection, endpoint-guided hybrid simulation, and UKF feasibility testing

Because the available CBP dataset is organized at the run level rather than as dense time-series trajectories, parameter estimation is primarily framed as an endpoint-learning problem. Mechanistic simulation is then used for dynamic reconstruction, while state estimation is examined only in a synthetic feasibility setting. Although the implemented workflow is endpoint-driven, the dynamic formulation is also discussed to show how the same grey-box structure could be extended when time-resolved CBP measurements become available. The relationship between the conceptual dynamic pipeline and the implemented endpoint-driven workflow is summarized in Fig. 2.

#### Dynamic approach (conceptual)

Phase-dependent kinetic targets could, in principle, be inferred directly from temporal data if sufficiently dense time-series measurements of biomass *X*(*t*), enzyme *E*(*t*), substrate *B*(*t*), soluble sugars *C*(*t*), and product *P*(*t*) were available. For example, after smoothing or regularized differentiation, approximate rate coefficients could be estimated as

14 $$\begin{aligned} \widehat{\mu }_1(t)&\approx \frac{1}{1-\frac{X(t)}{K}} \left( \frac{1}{X(t)}\frac{dX(t)}{dt}\right) +\mu _d \end{aligned}$$

and

15 $$\begin{aligned} \widehat{q}_E(t)&\approx \frac{1}{X(t)}\left( \frac{dE(t)}{dt}+k_{\deg }E(t)\right) . \end{aligned}$$

These approximate coefficients could then be learned using flexible nonlinear regressors. The resulting functions could be embedded within a hybrid state-space model as

16 $$\begin{aligned} \dot{\textbf{x}}&= f(\textbf{x},\boldsymbol{\theta },\textbf{u}) + \textbf{w}(t) \end{aligned}$$

and

17 $$\begin{aligned} \textbf{y}&= h(\textbf{x}) + \textbf{v}(t), \end{aligned}$$

where $\textbf{x} = [X, E, B, C, P]^\top$ denotes the latent state vector, consisting of biomass, enzyme, insoluble substrate, soluble sugar, and product; $\boldsymbol{\theta }$ collects the unknown kinetic and phase-transition parameters; and $\textbf{u}$ represents the exogenous operating conditions and time-invariant process descriptors. The function *f(· )* represents the nonlinear hybrid state-transition model, $\textbf{y}$ denotes the measured output vector, and $h(\textbf{x})$ maps the latent states to the measured variables. In a CBP monitoring context, the observation vector may contain only a subset of the internal states, such as biomass, soluble sugar, and scaled product measurements. The terms $\textbf{w}(t)$ and $\textbf{v}(t)$ represent process noise and measurement noise, respectively.

This conceptual formulation is provided to illustrate how the proposed endpoint-guided workflow could be extended if dense trajectory measurements become available. In this case, the dynamic rates could be directly estimated and then embedded in a hybrid state-space or soft-sensing framework, in line with broader nonlinear state-estimation and model-based monitoring approaches in biochemical processes (Alexander et al. 2020; Lyubenova et al. 2021).

#### Implemented endpoint-learning workflow

The implemented workflow uses a cross-sectional endpoint dataset consisting of 540 runs, 90 input features, and one output variable, the endpoint ethanol titer. Because the records are organized at the run level, endpoint learning was performed on the preprocessed encoded feature matrix rather than on time-resolved trajectories.

To assess the effect of response transformation, candidate endpoint models were evaluated using both logarithmically transformed and raw ethanol targets. Models with the suffix “_log” were trained with

18 $$\begin{aligned} z_i = \log (1+E_i), \end{aligned}$$

where $E_i$ is the endpoint ethanol titer for run *i*. Predictions from log-target models were mapped back to the ethanol-titer scale as

19 $$\begin{aligned} \widehat{E}_i = \exp (\widehat{z}_i)-1 . \end{aligned}$$

All RMSE, MAE, and $R^2$ values for log-target models were therefore computed on the inverse-transformed endpoint scale. Models with the suffix “_raw” were trained directly on $E_i$, allowing direct comparison between transformed and untransformed target formulations.

The endpoint dataset was refined using iterative out-of-fold (OOF) residual screening. A five-fold screening model was used to compute OOF residuals, and observations with the largest absolute residuals were removed in fixed *5~%* increments up to a maximum removal fraction of *25~%*. This step was used as a robustness-oriented data-curation procedure for the heterogeneous literature-derived corpus, not as evidence that removed records were experimentally invalid. Removed and retained samples, as well as the samples removed between the *10~%* and *15~%* screening levels, were examined using supplementary feature-distribution diagnostics.

The cleaned dataset was split into training and hold-out subsets. Random Forest, Extra Trees, histogram-based gradient boosting, XGBoost, LightGBM, and Gaussian-process regression were compared using both log-target and raw-target formulations where applicable (Breiman 2001; Geurts et al. 2006; Friedman 2001; Chen and Guestrin 2016; Ke et al. 2017; Rasmussen and Williams 2006). Model selection was based on repeated cross-validation on the training subset, while the hold-out subset was reserved for independent performance reporting. The selected endpoint surrogate was defined as

20 $$\begin{aligned} m_{\textrm{repCV}}^{*} = \arg \min _{m\in \mathcal {M}} \overline{\textrm{RMSE}}_{\textrm{repCV}}(m), \end{aligned}$$

where $\mathcal {M}$ denotes the full candidate-model set and $\overline{\textrm{RMSE}}_{\textrm{repCV}}(m)$ is the mean RMSE across repeated cross-validation folds. This strategy reduces dependence on a single random split and avoids using the hold-out set as the primary model-selection criterion (Kohavi 1995; Cawley and Talbot 2010; Varma and Simon 2006; Varoquaux 2018).

After model selection, the final endpoint surrogate was used for feature attribution, endpoint-guided hybrid calibration, and synthetic state-estimation analysis. Permutation importance and SHAP analysis were used to examine the selected surrogate within the cleaned feature space. Permutation importance quantifies the loss in predictive performance after feature randomization, whereas SHAP values provide additive local feature attributions that can be summarized across the dataset (Lundberg and Lee 2017; Molnar et al. 2024).

#### Endpoint-guided hybrid calibration

After endpoint-model selection, the final fitted surrogate was used to provide a representative endpoint target for the mechanistic hybrid simulator. The target was obtained by evaluating the selected endpoint model at the feature-wise median of the cleaned dataset. The mechanistic simulation was then evaluated under a representative operating condition: $50~{}^\circ \textrm{C}$, pH 5.5, pretreatment present, and an initial substrate concentration of $50~\mathrm {g\,L^{-1}}$. This condition was used as an illustrative benchmark within the range of the compiled CBP dataset, not as an optimized operating condition.

For each candidate multiplier vector $\boldsymbol{\alpha }$, the hybrid ordinary differential equation system was simulated over the batch horizon. The final simulated product was divided by a fixed scaling factor, $P_{\textrm{scale}}$, and compared with the surrogate-predicted endpoint as

21 $$\begin{aligned} S(\boldsymbol{\alpha }) = \left| \frac{P_{\boldsymbol{\alpha }}(t_f)}{P_{\textrm{scale}}} - \widehat{E}_{\textrm{end}} \right| , \end{aligned}$$

where $P_{\boldsymbol{\alpha }}(t_f)$, $P_{\textrm{scale}}$, and $\widehat{E}_{\textrm{end}}$ denote the simulated final product concentration, the product scaling factor, and the endpoint prediction from the selected surrogate model, respectively. The endpoint predictor therefore acts as an external calibration signal; it does not directly modify the mechanistic state equations.

The endpoint-guided search was carried out as a fixed-budget Monte Carlo sweep. The five phase-specific multipliers, *μ *, $q_E$, $V_{\max }$, $\mu _3$, and $Y_{P/X}$, were sampled independently from *0.8*–*1.2*. In total, 200 candidate parameter sets were simulated over a $72~\textrm{h}$ horizon and ranked by endpoint mismatch. The 20 solutions most closely aligned with the surrogate endpoint prediction were retained for dynamic analysis. Thus, the hybrid calibration is interpreted as endpoint-constrained dynamic reconstruction rather than full kinetic validation against time-resolved experimental data (Pérez et al. 2020; Agharafeie et al. 2023; Wang et al. 2023).

#### Synthetic unscented Kalman filter state-estimation setup

The accepted hybrid trajectories were used to generate noisy virtual measurements for testing an unscented Kalman filter (UKF). This step evaluates whether the endpoint-calibrated hybrid structure can support recursive reconstruction of internal states under partial observability (Rathore et al. 2021; Alexander et al. 2020; Yeboah et al. 2026c). The accepted endpoint-aligned trajectories were treated as surrogate “true” process realizations, controlled observation noise was added to selected states, and the UKF was used to reconstruct the latent dynamics.

This analysis is a synthetic feasibility test, not experimental soft-sensor validation. The measurements were generated from the same calibrated hybrid model family used for observer propagation, so the results assess numerical consistency and preliminary state-reconstruction feasibility under idealized conditions. This analysis is complementary to recent CBP digital-twin work in which observability, identifiability, sensor-set design, and UKF reconstruction were used to evaluate measurement packages for future CBP monitoring (Yeboah et al. 2026c). Overall, the implemented framework uses cross-sectional endpoint learning as the calibration signal, while mechanistic simulation and synthetic state estimation are used to recover plausible dynamics and assess downstream monitoring potential under data scarcity (Mahanty 2023; Jurinjak Tušek et al. 2024; Wang et al. 2023).

**Fig. 3:**
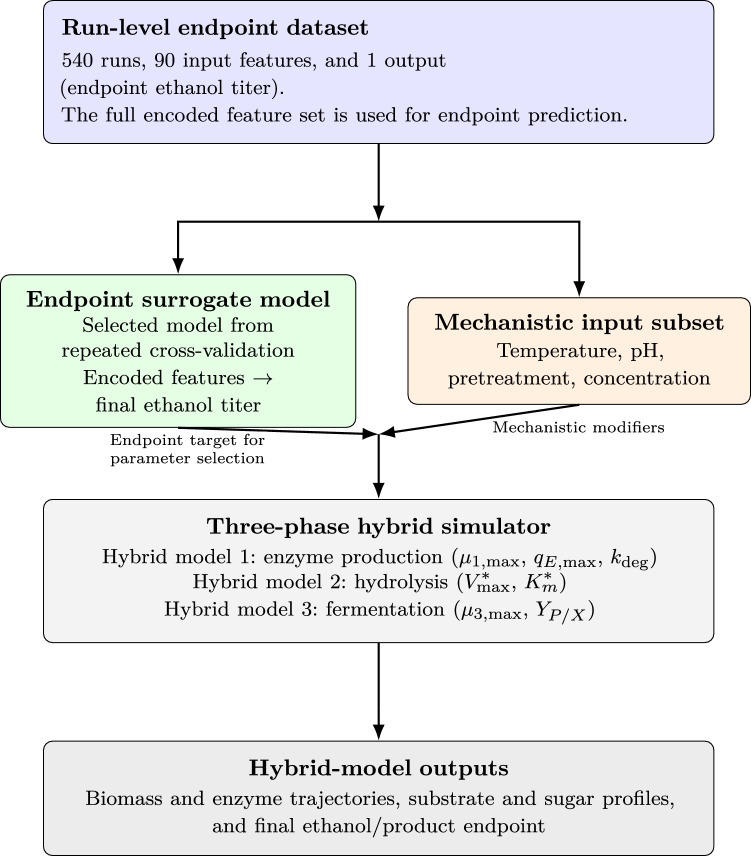
Data-to-model workflow for the endpoint-guided hybrid CBP framework. The full encoded run-level dataset is used for endpoint prediction, whereas temperature, pH, pretreatment, and concentration are passed as explicit mechanistic modifiers into the three-phase hybrid simulator. The surrogate-predicted endpoint is then used to guide parameter selection for the hybrid simulator and its resulting dynamic trajectories

The resulting data-to-model workflow is summarized in Fig. 3. The full encoded run-level covariates are used for endpoint prediction, whereas a mechanistically interpretable subset of operating descriptors, namely temperature, pH, pretreatment, and substrate concentration, is passed to the three-phase hybrid simulator. The selected endpoint surrogate then provides the calibration target used to guide the selection of endpoint-consistent dynamic parameter realizations.

### Model evaluation and verification

The framework was evaluated at three levels: endpoint-data curation, endpoint-surrogate performance, and synthetic state-estimation feasibility. The evaluation focused on residual diagnostics, endpoint prediction, feature attribution, endpoint-guided hybrid consistency, and unscented Kalman filter (UKF) feasibility, rather than trajectory-level experimental validation (Duong-Trung et al. 2023; Jurinjak Tušek et al. 2024; Alexander et al. 2020).

#### Evaluation of residual screening

The iterative five-fold out-of-fold (OOF) residual-screening procedure was evaluated using OOF RMSE, $R^2$, and the maximum absolute OOF residual at each removal stage. This step was treated as a data-curation diagnostic for the heterogeneous literature-derived dataset, not as evidence of external experimental generalization. Removed and retained samples were compared using supplementary feature-distribution diagnostics, and the samples removed between the *10~%* and *15~%* screening levels were analyzed separately because this interval produced a nonmonotonic change in OOF RMSE.

#### Endpoint-surrogate performance

After residual screening, the retained dataset was divided into training and hold-out subsets. Candidate raw-target and log-target endpoint models were evaluated using repeated cross-validation on the training subset. In the final workflow, 10 repeats of five-fold cross-validation were used, giving 50 train–validation evaluations per candidate model. The final endpoint surrogate was selected according to the lowest mean repeated-cross-validation RMSE on the training subset, while the hold-out subset was reserved for independent performance reporting. This separation was used to reduce selection bias and avoid using the hold-out set as the primary model-selection criterion (Kohavi 1995; Cawley and Talbot 2010; Varma and Simon 2006; Varoquaux 2018).

Hold-out performance was assessed using RMSE, MAE, and $R^2$:

22 $$\begin{aligned} \textrm{RMSE}&= \sqrt{\frac{1}{N}\sum _{i=1}^{N}(y_i-\hat{y}_i)^2}, \end{aligned}$$

23 $$\begin{aligned} \textrm{MAE}&= \frac{1}{N}\sum _{i=1}^{N}|y_i-\hat{y}_i|, \end{aligned}$$

24 $$\begin{aligned} R^2&= 1-\frac{\sum _{i=1}^{N}(y_i-\hat{y}_i)^2}{\sum _{i=1}^{N}(y_i-\bar{y})^2}, \end{aligned}$$

where $y_i$, $\hat{y}_i$, and $\bar{y}$ denote the observed endpoint, predicted endpoint, and mean observed endpoint, respectively. The selected endpoint surrogate was also evaluated before and after residual screening to separate the effect of data curation from the model structure itself.

#### Diagnostics, interpretability, and hybrid-model verification

The selected endpoint surrogate was examined using parity plots, residual-versus-predicted plots, residual histograms, repeated-cross-validation ranking, and hold-out diagnostics. Feature attribution was performed using permutation importance and SHAP analysis. Missingness indicators were excluded from substantive interpretation because they primarily reflect reporting patterns. Permutation importance summarizes global predictive dependence, whereas SHAP provides additive local feature contributions that can be summarized across the dataset (Lundberg and Lee 2017; Molnar et al. 2024). These results were interpreted as model-based process indicators rather than causal effects.

The hybrid simulator was evaluated by ranking candidate parameter sets according to endpoint mismatch with the selected surrogate prediction. Accepted solutions were then examined using phase weights, endpoint alignment, and trajectory envelopes. Because measured dynamic trajectories were unavailable for most records, this step assessed endpoint consistency and mechanistic plausibility, not trajectory-level experimental accuracy.

#### State-estimation verification metrics

State-estimation feasibility was assessed using a UKF driven by synthetic measurements generated from accepted endpoint-calibrated hybrid trajectories. Zero-mean Gaussian noise was added to selected measured states, and a backward smoothing pass was tested as a post-processing step. The UKF analysis was therefore treated as a synthetic feasibility test, not as experimental soft-sensor validation.

The UKF performance was quantified using RMSE, MRPE, MRPU, and $R^2$. The RMSE and $R^2$ follow Eqs. (22) and (24), whereas MRPE and MRPU are defined as

25 $$\begin{aligned} \textrm{MRPE}&= \frac{1}{N}\sum _{i=1}^{N} \frac{|y_i-\hat{y}_i|}{\max (|\hat{y}_i|,\epsilon )}\times 100~\%, \end{aligned}$$

26 $$\begin{aligned} \textrm{MRPU}&= \frac{1}{N}\sum _{i=1}^{N} \frac{\sigma _i}{\max (|\hat{y}_i|,\epsilon )}\times 100~\%, \end{aligned}$$

where $y_i$, $\hat{y}_i$, $\sigma _i$, and *ε * denote the reference value, UKF estimate, estimated uncertainty magnitude, and a small positive constant used to prevent numerical inflation near zero, respectively (Rathore et al. 2021; Gallego et al. 2020; Schweidtmann et al. 2024; Alexander et al. 2020).

The mechanistic, data-driven, and coupling elements of the endpoint-guided grey-box framework are summarized in Table 2.

**Table 2:** Summary of mechanistic, data-driven, and coupling elements in the endpoint-guided grey-box framework

Component	Type	Role
Phase weights $\phi _1(t)$–$\phi _3(t)$	Mechanistic / structural	Smoothly switch between enzyme production, hydrolysis, and fermentation.
State equations	Mechanistic	Describe biomass, enzyme, substrate, sugar, and product dynamics.
Nominal coefficients and modifiers	Semi-mechanistic	Encode prior kinetic knowledge and condition dependence.
Endpoint predictor and model selection	Data-driven	Learn endpoint ethanol titer and select the final surrogate using repeated cross-validation on the training subset.
Hold-out evaluation	Verification / reporting	Report independent endpoint-prediction performance without using the hold-out subset for model selection.
Endpoint-guided parameter search	Coupling layer	Retain mechanistic parameter sets consistent with the selected surrogate endpoint.
Synthetic UKF study	Downstream verification	Test state-estimation feasibility using calibrated hybrid trajectories and model-generated noisy measurements.

### Computational reproducibility

The analyses were implemented in Python 3.13.5 within Visual Studio Code using a fixed random seed of 42. The workflow used numpy 2.3.2, pandas 2.3.1, matplotlib 3.10.3, scipy 1.16.0, scikit-learn 1.7.1, joblib 1.5.1, lightgbm 4.6.0, xgboost 3.1.1, shap 0.49.1, and filterpy 1.4.5.

## Results and discussion

### Dataset and target characterization

The dataset consists of 540 literature-derived CBP runs, represented by 90 input variables and one output variable, the endpoint ethanol titer. The input space covers physicochemical, biological, and operational descriptors, including temperature, pH, pretreatment-related variables, substrate concentration, mixing conditions, and encoded microbial-consortium characteristics. This dataset is broader and more feature-rich than earlier CBP machine-learning studies; for example, Yeboah et al. (2024) used 96 literature-derived records, later reduced to 50 refined records after residual-based cleaning, with 11 input variables and one bioethanol-yield output. The expanded feature representation used in the present work was designed to capture a wider range of feedstock, pretreatment, operating, and microbial descriptors while preserving the endpoint-focused structure of the available literature data.

The observed endpoint values vary substantially across studies, reflecting considerable heterogeneity in feedstock properties, microbial compositions, reporting practices, analytical methods, and operating conditions. This variability allows the dataset to support surrogate endpoint modeling across a broad range of CBP settings, but it also requires careful interpretation because the records were compiled from the literature rather than generated in a single controlled experimental campaign. Such heterogeneity is expected in CBP because ethanol formation depends on the combined effects of biomass accessibility, enzyme production, hydrolysis, sugar utilization, and fermentation rather than on a single process variable (Brethauer and Studer 2014; Ahamed et al. 2021). Experimental CBP studies have also shown that pretreatment, substrate composition, organism choice, pH, temperature, and process configuration can strongly influence ethanol production (Kavitha et al. 2022; Yeboah et al. 2024).

Spearman’s rank analysis indicates that the endpoint is highly multifactorial. The strongest monotonic associations mainly involve temperature and microbial-consortium descriptors, followed by pH and mixing rate, as summarized in Table 3. In the compiled dataset, negative coefficients for temperature, pH, bacteria-associated indicators, thermophilic status, and strict anaerobicity are associated with lower endpoint ethanol titers. In contrast, positive coefficients for yeast-related indicators, mixing rate, enzyme-assisted pretreatment, and substrate concentration are associated with higher titers. These directions are broadly consistent with the expectation that ethanol production is sensitive to the operating environment and microbial phenotype. However, the signs should be interpreted as dataset-level associations rather than universal biological rules. For example, the negative association for temperature does not imply that lower temperature is always preferable for CBP; rather, it reflects how temperature is confounded in the compiled records with thermophilic organisms, bacterial systems, anaerobic operation, feedstock type, and study-specific protocols. Similarly, the positive yeast-related associations are consistent with the strong ethanologenic role of yeast-based systems, but they should not be taken as evidence that yeast-only CBP is universally superior to bacterial or mixed-consortium systems.

The narrow bootstrap *95~%* confidence intervals and very small *p*-values for the top variables suggest that these ranked associations are robust within the compiled dataset. Nevertheless, these correlations should be interpreted as descriptive associations rather than causal effects. Some microbial descriptors are overlapping indicator features, and many process variables are likely confounded by organism choice, feedstock type, and study-specific operating protocols. Overall, these results provide an exploratory overview of the most prevalent monotonic directions in the compiled CBP literature dataset (Table 3). A complete catalogue of the encoded endpoint-learning features, including variable type, unit or encoding, observed range, and definition, is provided in Table S2 of the Supplementary Materials, while only the top-ranked features are shown here for brevity.

**Table 3:** Top features ranked by absolute Spearman correlation with the endpoint ethanol titer. Confidence intervals (CIs) are*95~%*bootstrap intervals for*|ρ |*

Feature	*|ρ |*	*95~%*CI for*|ρ |*	*ρ *	*p*-value
Temperature (${}^\circ \textrm{C}$)	0.529	[0.464, 0.592]	*-0.529*	$2.88\times 10^{-40}$
Bacteria-only consortium	0.512	[0.452, 0.570]	*-0.512*	$2.23\times 10^{-37}$
Presence of bacteria	0.509	[0.449, 0.567]	*-0.509*	$5.82\times 10^{-37}$
Presence of yeast	0.476	[0.408, 0.538]	0.476	$7.34\times 10^{-32}$
Thermophilic microorganism present	0.475	[0.402, 0.540]	*-0.475*	$9.56\times 10^{-32}$
Yeast-only consortium	0.471	[0.397, 0.537]	0.471	$4.10\times 10^{-31}$
pH	0.469	[0.392, 0.538]	*-0.469*	$7.84\times 10^{-31}$
Mixing rate ($\textrm{rpm}$)	0.425	[0.355, 0.496]	0.425	$3.72\times 10^{-25}$
Strict anaerobe present	0.381	[0.306, 0.448]	*-0.381*	$4.70\times 10^{-20}$
Enzyme-assisted pretreatment	0.366	[0.295, 0.431]	0.366	$1.60\times 10^{-18}$

### Out-of-fold residual trimming and its effect on endpoint prediction

Before final model training, the ethanol dataset was subjected to iterative out-of-fold (OOF) residual trimming to improve robustness against anomalous endpoint observations. At each stage, a five-fold OOF screening model was used to predict samples that were not included during fitting. Residuals were then computed, and the observations with the largest absolute residuals were removed in fixed increments. The purpose of this procedure was to reduce the influence of highly inconsistent records in the heterogeneous literature-derived dataset while limiting direct leakage from the fitted model into the residuals used for screening. This step is consistent with the need for careful curation of literature-derived bioprocess datasets, where variation in feedstock, organism, protocol, analytical method, and reporting quality can introduce substantial noise (Fisher et al. 2022; Huntington et al. 2023; Jurinjak Tušek et al. 2024). It also extends earlier CBP machine-learning work, in which residual-based cleaning was used to improve prediction from a smaller literature-derived dataset (Yeboah et al. 2024). The trimming path is summarized in Table 4.

A substantial reduction in prediction error was observed after the first two trimming steps. The OOF RMSE decreased from 6.450 in the untrimmed dataset to 3.275 after *5~%* removal and to 2.698 after *10~%* removal. The trimming trajectory was not strictly monotonic: at the *15~%* removal stage, the OOF RMSE increased to 5.510 and the maximum absolute residual increased to 65.597. This temporary deterioration indicates that the observations removed between the *10~%* and *15~%* stages contained information that strongly affected the screening model. After further trimming, however, the OOF RMSE decreased again to 1.885 at *20~%* removal and to 1.819 at *25~%* removal. The recovery in performance at the 20~\% trimming level suggests that the retained observations were not simply less affected by outliers, but also preserved informative heterogeneous samples that contributed to model robustness.The final retained dataset, obtained after removing *25~%* of the original observations, therefore achieved the lowest OOF RMSE and a high OOF coefficient of determination of $R^2=0.961$. At this stage, the maximum absolute OOF residual had also decreased markedly relative to the untrimmed dataset, suggesting that the retained subset was more internally consistent.

The nonmonotonic behavior at the *15~%* stage shows that residual trimming should be viewed as a robustness-oriented curation procedure rather than a guaranteed monotonic optimization process. Because the screening model is refitted after each removal step, the residual structure can change when influential observations are removed. Therefore, the removed observations should not be regarded as incorrect experimental results. Instead, they are treated as high-disagreement records under the selected endpoint-learning representation. To address this issue directly, the samples removed between the *10~%* and *15~%* screening levels were analyzed separately. In this sense, the trimming procedure is not a simple outlier deletion step, but a deliberate curation strategy that seeks to reduce internally inconsistent records while retaining useful variation within the heterogeneous CBP dataset. This interval-specific analysis compared the removed samples with the final retained subset using standardized mean differences and feature-distribution plots. The largest shifts involved operating and categorical descriptors such as temperature, substrate concentration, feedstock class, yeast-only and bacteria-only indicators, mixing rate, thermophilic status, and pretreatment-class variables. These shifts indicate that the *10*–*15~%* removed group was not a random subset of the data but rather represented a distinct region of the heterogeneous CBP feature space. Thus, the temporary increase in OOF RMSE is consistent with the removal of influential samples that may represent valid but underrepresented experimental regimes rather than only obvious experimental outliers.

**Fig. 4:**
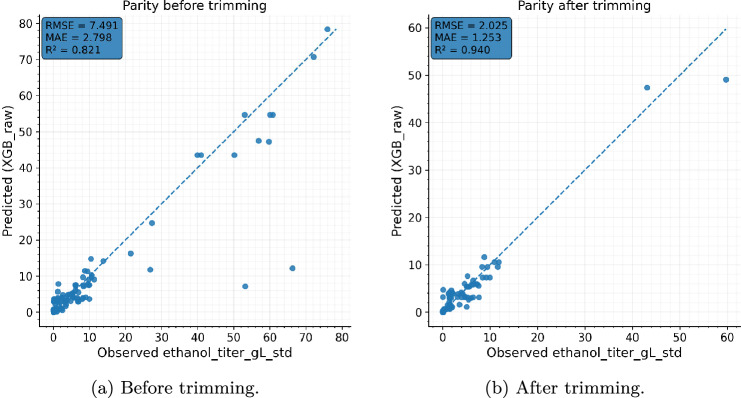
Hold-out parity plots for the repeated-cross-validation-selected endpoint model, XGB_raw, before and after out-of-fold residual trimming. Trimming tightened agreement around the identity line and reduced large endpoint deviations

**Fig. 5:**
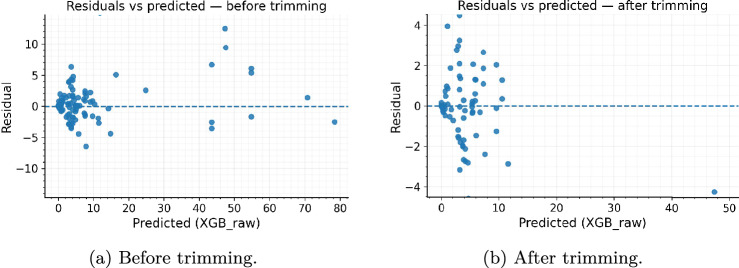
Residual-versus-predicted diagnostics for the repeated-cross-validation-selected endpoint model, XGB_raw, before and after out-of-fold residual trimming. The trimmed dataset produced a narrower and more centered residual distribution

The hold-out parity and residual diagnostics further illustrate the effect of trimming for the repeated-cross-validation-selected endpoint model, XGB_raw. Before trimming, the parity plot showed considerable scatter at moderate and high endpoint values, with several clear cases of underprediction and overprediction (Fig. 4a). After trimming, the predictions were more tightly clustered around the identity line, and the hold-out error metrics improved (Fig. 4b). The residual-versus-predicted plots show the same pattern: before trimming, the residuals were more widely dispersed and included several large positive deviations, whereas after trimming the residual cloud became narrower and more centered around zero (Fig. 5a, b).

**Table 4:** Summary of iterative out-of-fold residual trimming

Stage	*n*	Removed	Removed (%)	Max *|*residual*|*	OOF RMSE/$R^2$
1	540	0	0	54.619	6.450/0.799
2	513	27	5	18.427	3.275/0.942
3	486	54	10	17.272	2.698/0.952
4	459	81	15	65.597	5.510/0.785
5	432	108	20	15.008	1.885/0.963
6	405	135	25	10.769	1.819/0.961

To further characterize the residual-screening step, the feature distributions of retained and removed observations were compared. The removed subset showed clear shifts in several key variables. For example, substrate concentration was substantially higher in the removed samples, with a mean of $101.194~\mathrm {g\,L^{-1}}$, compared with $29.151~\mathrm {g\,L^{-1}}$ in the retained samples. In contrast, temperature was lower in the removed subset, with a mean of $35.081~{}^\circ \textrm{C}$, compared with $46.001~{}^\circ \textrm{C}$ in the retained subset. The pH also shifted downward, from a retained-sample mean of 7.345 to a removed-sample mean of 6.353. Categorical indicators showed similar differences: bacteria-only systems were more common in the retained subset, whereas yeast-only systems and other feedstock classes were more frequent among the removed samples. These results indicate that the removed observations were not randomly distributed but were concentrated in regions of the heterogeneous literature-derived feature space with larger endpoint inconsistency, as shown in Supplementary Table S3. The additional *10*–*15~%* interval diagnostics are reported separately in the Supplementary Materials to make the nonmonotonic trimming stage transparent.

Overall, OOF residual trimming improved the internal consistency of the literature-derived dataset and produced a cleaner modeling subset for subsequent endpoint-model comparison. The final trimmed dataset was therefore used for repeated-cross-validation model selection, hold-out reporting, feature-importance analysis, SHAP interpretation, and endpoint-guided hybrid simulation. However, the removed- and interval-sample diagnostics show that trimming should be interpreted as a robustness-oriented curation step rather than as evidence that the removed records were experimentally incorrect. This conclusion should also be understood in the context of the compiled dataset: trimming improves internal predictive consistency, but it is not a substitute for external validation using independently generated CBP endpoint measurements or time-resolved CBP trajectories.

### Endpoint prediction performance

The trimmed cross-sectional ethanol dataset was used for endpoint prediction after iterative out-of-fold residual screening and model comparison. The model set included Random Forest (RF), Extra Trees (ET), histogram-based gradient boosting (HGB), XGBoost (XGB), LightGBM (LGBM), and Gaussian-process (GP) regression. To assess the effect of target transformation, candidate models were evaluated using both log-transformed and raw endpoint targets where applicable. Models with the suffix _log were trained on the transformed target *łog (1+E)*, whereas models with the suffix _raw were trained directly on the ethanol endpoint. This model set provided an internal endpoint-prediction benchmark across bagging-based ensembles, randomized tree ensembles, boosting methods, and probabilistic kernel regression (Breiman 2001; Geurts et al. 2006; Friedman 2001; Chen and Guestrin 2016; Ke et al. 2017; Rasmussen and Williams 2006).

Candidate models were first tuned and ranked using cross-validated RMSE on the cleaned training subset. Because model rankings from a single cross-validation run or a single hold-out split can be unstable, the final model-selection step was based on repeated cross-validation. Specifically, all raw-target and log-target candidate models were evaluated using 10 repeats of five-fold cross-validation on the training subset. The hold-out subset was reserved for independent reporting and was not used as the primary selection criterion. This evaluation design was used to reduce dependence on a single random split and to avoid overinterpreting small differences between model families (Kohavi 1995; Cawley and Talbot 2010; Varma and Simon 2006; Varoquaux 2018).

The repeated-cross-validation ranking is summarized in Table 5. The lowest mean repeated-cross-validation RMSE was obtained by XGB_raw, with $\textrm{RMSE}=1.566 \pm 0.465$. This model was therefore selected as the final endpoint surrogate for feature-attribution analysis, endpoint-guided hybrid calibration, and synthetic UKF feasibility analysis. The next-best models were HGB_log and HGB_raw, with repeated-cross-validation RMSE values of *1.593 ± 0.559* and *1.601 ± 0.493*, respectively. The small differences among these leading boosting-based models, relative to the repeated-cross-validation variability, indicate that the exact top-ranked model is sensitive to the evaluation criterion. Therefore, the results are interpreted as evidence that boosting-based tree ensembles are the most robust endpoint-prediction family for the curated CBP dataset, rather than as evidence that one specific model is universally superior.

The independent hold-out comparison is shown in Table 6. Although XGB_raw was selected by repeated cross-validation, the hold-out subset favored the histogram-based gradient-boosting models. In particular, HGB_log achieved the lowest hold-out RMSE of 1.540 and the highest hold-out $R^2=0.965$, followed closely by HGB_raw. In contrast, the selected XGB_raw model achieved hold-out RMSE *=2.025*, MAE *=1.253*, and $R^2=0.940$. This discrepancy between repeated-cross-validation selection and hold-out ranking further supports the conclusion that the leading boosting-based models are closely matched and that final model ranking depends on the validation design.

The selected XGB_raw model also showed a clear improvement after out-of-fold residual screening. Before trimming, the same model class and specification gave hold-out RMSE *=7.491*, MAE *=2.798*, and $R^2=0.821$. After trimming, hold-out performance improved to RMSE *=2.025*, MAE *=1.253*, and $R^2=0.940$, as summarized in Table 7. These results support the role of the residual-screening stage in improving internal predictive consistency within the curated literature-derived dataset. However, this improvement should be interpreted as internal generalization within the compiled corpus, not as external validation on independently generated CBP experiments.

This finding can also be compared with the earlier CBP machine-learning study by Yeboah et al. (2024), in which Gaussian-process regression gave the best performance for a smaller literature-derived CBP dataset with microbial consortia. In that study, 96 experimental records were collected from the literature and reduced to 50 refined records after residual-based cleaning, with 11 input variables used for endpoint prediction. In contrast, the present work uses a larger 540-run dataset with 90 encoded input features, including broader representations of feedstock, pretreatment, operating, microbial, and missingness descriptors. Therefore, the lower ranking of Gaussian-process regression in the present model set should not be viewed as a contradiction of the earlier study. Rather, it indicates that the preferred endpoint surrogate depends on the data regime: Gaussian-process regression can perform well for smaller and more compact datasets, whereas boosting-based tree ensembles can be more effective for larger, heterogeneous tabular datasets with mixed variable types.

Overall, the endpoint-learning phase is better described as a model-selection and sensitivity-comparison paradigm than as a Gaussian-process-centric surrogate pipeline. The raw-target comparison confirms that target transformation should be checked empirically because the final repeated-cross-validation-selected model was XGB_raw. At the same time, the strong hold-out performance of HGB_log and HGB_raw shows that the most defensible conclusion is at the model-family level: boosting-based tree ensembles provided the most robust endpoint predictors for the curated CBP dataset. The selected XGB_raw surrogate was used for permutation importance, SHAP interpretation, endpoint-guided hybrid simulation, and synthetic UKF analysis to keep the downstream workflow internally consistent. This interpretation is limited to generalization within the curated literature-derived corpus because no additional external endpoint-validation dataset was available.

**Table 5:** Repeated cross-validation ranking for endpoint prediction on the trimmed training set. Repeated cross-validation used 10 repeats of five-fold cross-validation. Lower mean repeated-cross-validation RMSE indicates better performance

Model	Target	Repeated-CV RMSE	Rank
XGB_raw	Raw	*1.566 ± 0.465*	1
HGB_log	Log	*1.593 ± 0.559*	2
HGB_raw	Raw	*1.601 ± 0.493*	3
ET_log	Log	*1.634 ± 0.554*	4
ET_raw	Raw	*1.672 ± 0.725*	5
XGB_log	Log	*1.673 ± 0.530*	6
RF_log	Log	*1.726 ± 0.528*	7
RF_raw	Raw	*1.750 ± 0.589*	8
LGBM_log	Log	*1.752 ± 0.624*	9
LGBM_raw	Raw	*1.850 ± 1.116*	10
GP_raw (RBF+MAT, $\alpha = 3\times 10^{-4}$)	Raw	*2.141 ± 0.772*	11
GP_log (RBF+MAT, $\alpha = 10^{-4}$)	Log	*2.623 ± 1.496*	12

**Table 6:** Independent hold-out performance for all raw-target and log-target candidate models after trimming. The hold-out subset was used for reporting, not as the primary model-selection criterion

Model	Hold-out RMSE	Hold-out $R^2$
HGB_log	1.540	0.965
HGB_raw	1.552	0.965
LGBM_log	1.594	0.963
XGB_log	1.611	0.962
LGBM_raw	1.713	0.957
RF_raw	1.778	0.953
RF_log	1.814	0.951
ET_raw	1.906	0.946
ET_log	1.913	0.946
XGB_raw	2.025	0.940
GP_raw (RBF+MAT, $\alpha = 3\times 10^{-4}$)	2.353	0.918
GP_log (RBF+MAT, $\alpha = 10^{-4}$)	3.069	0.861

**Table 7:** Hold-out performance of the repeated-cross-validation-selected endpoint model, XGB_raw, before and after out-of-fold residual trimming

Dataset version	RMSE	MAE	$R^2$
Before trimming hold-out	7.491	2.798	0.821
After trimming hold-out	2.025	1.253	0.940

### Determinants of endpoint variability

#### Permutation importance

Permutation importance was calculated for the final repeated-cross-validation-selected endpoint surrogate, XGB_raw, using the trimmed hold-out set. Missingness-indicator variables were excluded from substantive interpretation (Kaneko 2022; Molnar et al. 2024). Because permutation importance measures the reduction in predictive performance after randomizing a feature, the results should be interpreted as model-based predictive dependencies rather than as direct causal effects.

**Fig. 6:**
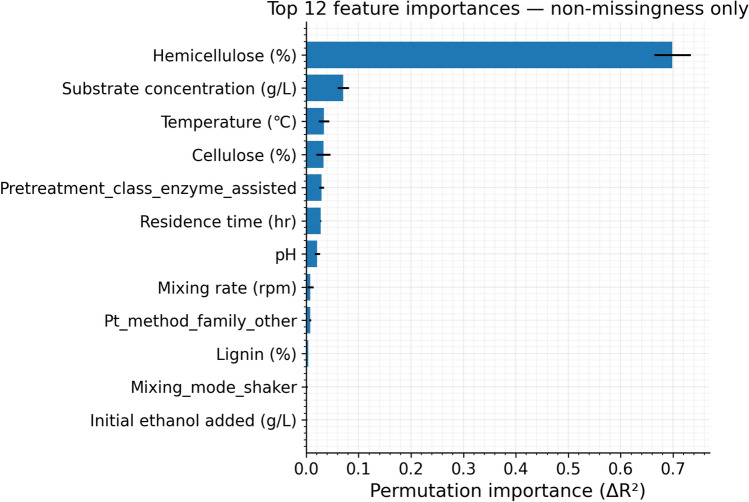
Top 12 permutation feature importances for the final repeated-cross-validation-selected XGB_raw endpoint model on the trimmed hold-out set, excluding missingness-indicator variables. Error bars denote variability across repeated permutations

The ranking indicates that endpoint ethanol titer is mainly influenced by feedstock composition and substrate-accessibility variables, followed by operating descriptors. The most influential feature was hemicellulose content, which produced the largest decrease in predictive performance when permuted, as shown in Fig. 6. A second tier of importance was formed by substrate concentration, temperature, cellulose content, enzyme-assisted pretreatment, residence time, pH, and mixing rate. These results indicate that the selected XGB_raw model relies strongly on substrate composition, substrate loading, thermal conditions, and pretreatment-related accessibility when predicting endpoint ethanol titer. This interpretation is consistent with the CBP process context, where feedstock accessibility, pretreatment, enzyme activity, hydrolysis, sugar release, and fermentation jointly determine final ethanol production (Brethauer and Studer 2014; Kavitha et al. 2022; Sharma et al. 2022). It also agrees with earlier CBP machine-learning work, where substrate composition, temperature, pH, residence time, pretreatment, and substrate concentration were treated as important endpoint-prediction variables, although within a smaller 96-record dataset that was later refined to 50 observations (Yeboah et al. 2024).

Microbial descriptors and process-configuration variables contributed less in the permutation ranking than substrate-composition and operating variables. This pattern does not imply that microbial properties are unimportant for CBP. Rather, it suggests that, in the selected XGB_raw endpoint model, much of the hold-out predictive loss was driven by variables directly linked to feedstock composition, substrate availability, and the operating environment. Microbial effects may also be distributed across overlapping encoded indicators, organism categories, and process descriptors, which can reduce the apparent importance of any single microbial feature. Similarly, pH and residence time appeared as secondary modifiers rather than primary drivers in the permutation ranking, but they remain biologically relevant because they affect enzyme activity, microbial growth, hydrolysis, and fermentation efficiency.

As an additional robustness check, permutation importance was also computed before trimming and compared with the post-trimming values. Substrate concentration remained influential in both analyses, although its importance decreased from *0.429 ± 0.041* before trimming to *0.071 ± 0.011* after trimming. Temperature showed the opposite trend, increasing from *0.020 ± 0.002* before trimming to *0.034 ± 0.010* after trimming. Hemicellulose became the dominant post-trimming predictor, increasing from *0.005 ± 0.003* before trimming to *0.699 ± 0.035* after trimming. Several categorical indicators showed much lower importance after residual screening; for example, the yeast-only indicator decreased from *0.013 ± 0.004* to approximately zero, and the bacteria-associated indicator decreased from *0.011 ± 0.002* to *0.001 ± 0.001*. These changes suggest that trimming reduced the influence of dataset-specific categorical heterogeneity while retaining the main process-relevant drivers, especially substrate composition, substrate loading, and temperature, as shown in Supplementary Table S4.

Overall, the permutation-importance results indicate that hemicellulose, substrate loading, temperature, cellulose, enzyme-assisted pretreatment, residence time, pH, and mixing form the dominant predictive structure of the final endpoint model. These results support the interpretation that endpoint ethanol variability in the curated literature-derived dataset is controlled primarily by feedstock composition, substrate accessibility, and operating conditions, with microbial descriptors acting as additional modifiers.

#### Shapley additive explanations analysis

**Fig. 7:**
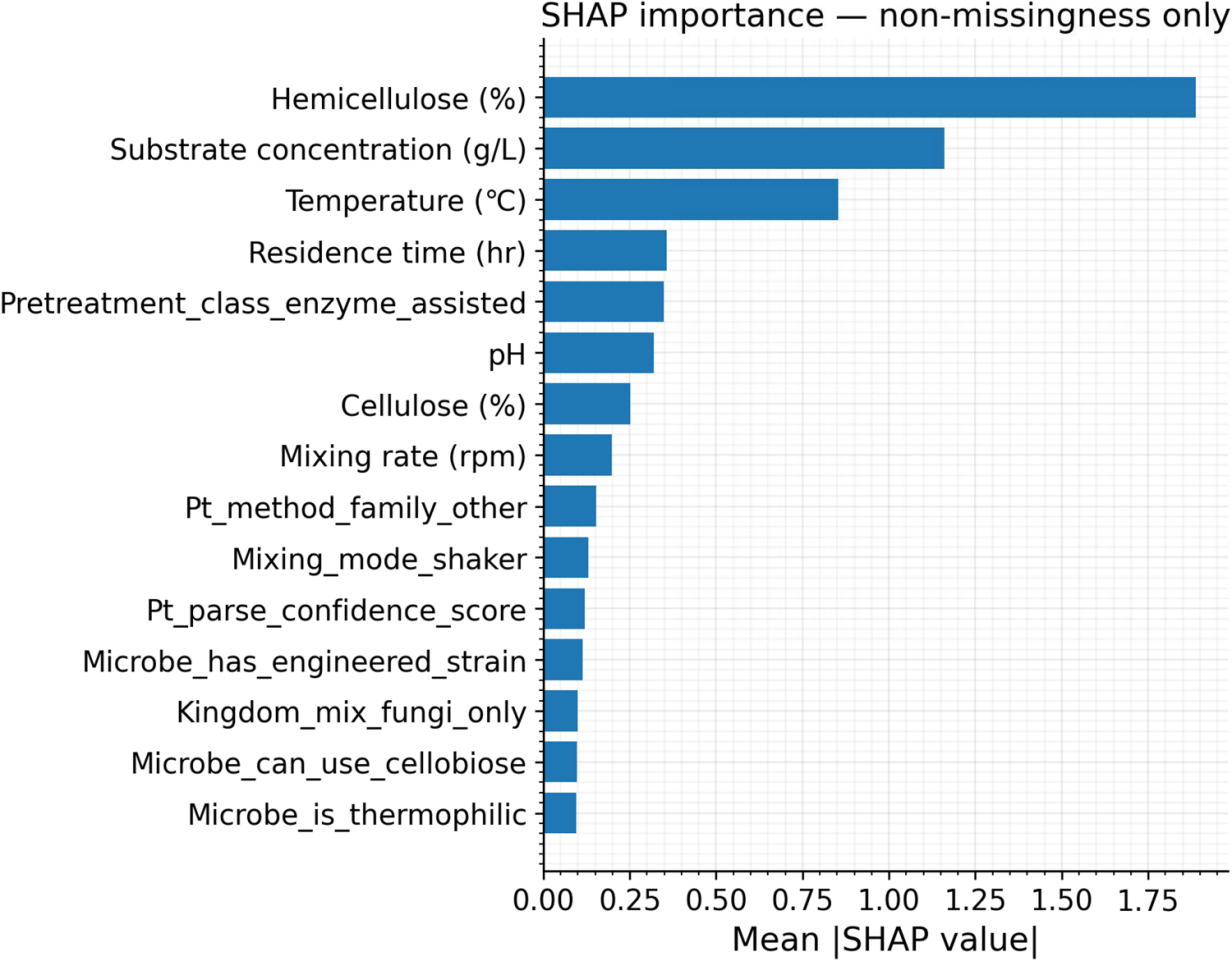
Global SHAP importance for the final repeated-cross-validation-selected XGB_raw endpoint model, computed using non-missingness features only. Bars represent the mean absolute SHAP value for each feature

Shapley additive explanations (SHAP) were used to examine both global feature importance and local response patterns of the final repeated-cross-validation-selected XGB_raw endpoint model. Missingness-indicator variables were excluded from interpretation. SHAP decomposes individual model predictions into additive feature contributions, making it useful for interpreting nonlinear ensemble models at both local and global levels (Lundberg and Lee 2017; Molnar et al. 2024). For the selected XGBoost surrogate, SHAP values were computed using the native XGBoost contribution method. The global SHAP ranking indicates that hemicellulose content is the most influential predictor of endpoint ethanol titer, followed by substrate concentration and temperature, as shown in Fig. 7. Residence time, enzyme-assisted pretreatment, pH, cellulose content, and mixing rate form a second tier of influence.

The prominence of hemicellulose and substrate concentration in the SHAP ranking is consistent with the role of feedstock composition and substrate loading in CBP performance. Hemicellulose can affect biomass accessibility and the availability of soluble sugars, whereas substrate concentration determines the amount of fermentable material available to the system. However, these variables should not be interpreted as simple monotonic optimization levers. High substrate loadings can improve potential ethanol production, but they may also introduce mass-transfer limitations, inhibition, or reduced accessibility. Similarly, hemicellulose effects depend on feedstock type, pretreatment history, hydrolysis efficiency, and the ability of the microbial system to utilize released sugars.

Temperature also ranked highly in the SHAP analysis. This result does not mean that temperature alone universally determines ethanol formation. Rather, temperature acts as an organizing variable in the compiled literature dataset because it is closely linked with organism class, thermophilic status, enzyme activity, fermentation regime, and study-specific operating protocols. This interpretation is consistent with CBP studies showing that thermal conditions influence both microbial metabolism and hydrolytic activity, while also interacting with pH, substrate loading, and pretreatment severity (Yeboah and Söffker 2026; Brethauer and Studer 2014; Sharma et al. 2022). The high ranking of substrate concentration and enzyme-assisted pretreatment similarly supports the view that endpoint ethanol titer is strongly constrained by upstream accessibility and sugar-release potential, not only by downstream fermentation capacity.

Additional SHAP dependence plots for hemicellulose content, pH, mixing rate, and residence time are provided in Supplementary Figures 1–4. These plots show nonlinear and interacting feature effects. Hemicellulose exhibits a strong positive contribution at higher values in the fitted endpoint model, whereas lower hemicellulose values are associated with weak or negative SHAP contributions. The pH response is nonlinear, consistent with the biological expectation that enzyme activity and microbial fermentation are favored within limited pH ranges rather than at extreme values. Mixing rate also shows a context-dependent contribution, which is consistent with the role of agitation in reducing transport limitations while interacting with reactor configuration and feedstock properties. Residence time contributes in a context-dependent manner, reflecting the fact that longer residence time may increase conversion only when hydrolysis and fermentation conditions are otherwise favorable. These patterns agree with experimental and data-driven CBP studies in which operating conditions and feedstock descriptors jointly affect endpoint ethanol production (Kavitha et al. 2022; Yeboah et al. 2024).

The dependence plots should not be treated as direct optimization rules because the compiled data are heterogeneous and many variables are confounded by feedstock type, organism choice, pretreatment method, and reporting practice. For example, a positive SHAP contribution at a specific hemicellulose content or substrate concentration does not imply that independently increasing that feature will always improve CBP performance. Similarly, temperature and pH effects should be interpreted as dataset-level model responses rather than as universal operating optima.

Together, the SHAP and permutation-importance analyses support the interpretation that endpoint ethanol production is governed by interacting compositional, thermal, and operational factors. Hemicellulose, substrate concentration, temperature, residence time, pretreatment, pH, cellulose content, and mixing form the main predictive structure of the final XGB_raw endpoint model. These feature-attribution results are consistent with the CBP process context, in which temperature, pH, feedstock accessibility, substrate concentration, pretreatment, enzyme formation, hydrolysis, and fermentation efficiency jointly determine ethanol production (Yeboah and Söffker 2026; Cheng et al. 2023; Ahamed et al. 2021).

More broadly, these feature-attribution results are consistent with recent dynamic optimization results for CBP. Yeboah et al. (2026b) showed that CBP operation involves trade-offs among ethanol titer, productivity, substrate conversion, soluble sugar accumulation, operating severity, control movement, and batch duration. In that study, time-varying temperature and pH policies were needed to explore feasible trade-off regions that could not be represented by a single static operating condition. The present results support the same interpretation from a data-driven endpoint-modeling perspective: endpoint ethanol performance is not governed by one variable alone but by the coupled effects of substrate accessibility, pretreatment history, feedstock composition, microbial and process configuration, and the operating environment.

### Endpoint-guided hybrid model identification

The parameter set selected by the endpoint-guided search under the representative operating condition of $50~{}^\circ \textrm{C}$, pH 5.5, pretreatment present, and initial solids of $50~\mathrm {g\,L^{-1}}$ is summarized in Table 8. This operating point was chosen as a plausible benchmark condition within the range of the compiled CBP dataset, rather than as an experimentally optimized condition. It provides a controlled reference case for illustrating how the endpoint surrogate can guide dynamic reconstruction when time-resolved measurements are unavailable.

The best solution was obtained using the final repeated-cross-validation-selected XGB_raw endpoint model, which provided a surrogate target of 2.6473. Although the independent hold-out subset favored the HGB models, the downstream hybrid analysis was kept internally consistent with the repeated-cross-validation-selected endpoint surrogate. Therefore, feature attribution, endpoint-guided calibration, and synthetic state-estimation analysis were all based on the same XGB_raw model.

Only moderate increases relative to the nominal baseline were required: $\textrm{Scale}_{\mu }=1.1856$, $\textrm{Scale}_{q_E}=1.1263$, $\textrm{Scale}_{V_{\max }}=1.0894$, $\textrm{Scale}_{\mu _3}=1.1476$, and $\textrm{Scale}_{Y_P}=1.1627$. This pattern suggests that endpoint agreement was achieved through slightly enhanced growth, enzyme production, hydrolysis, and downstream product formation, rather than through extreme kinetic distortion. This interpretation is consistent with the general CBP process view that ethanol formation is governed by the coupling of enzyme production, substrate hydrolysis, sugar availability, microbial growth, and fermentation. Experimental CBP studies emphasize that efficient ethanol production requires coordinated hydrolysis and fermentation under suitable organism and operating conditions (Brethauer and Studer 2014; Kavitha et al. 2022). Similarly, mechanistic CBP modeling studies have shown that cellulose depolymerization, enzyme regulation, and fermentative conversion are strongly coupled and should not be interpreted as isolated subprocesses (Ahamed et al. 2021). Therefore, the moderate simultaneous adjustment of growth, enzyme production, hydrolysis, and fermentation coefficients in the present endpoint-guided solution is biologically reasonable.

Under the intended grey-box interpretation, the corresponding phase-specific parameters are biologically plausible. In the enzyme-production phase, $\mu =0.2371~\textrm{h}^{-1}$, $\mu _d=0.0100~\textrm{h}^{-1}$, $q_E=0.0743~\mathrm {U\,g^{-1}\,h^{-1}}$, and $k_{\textrm{deg}}=0.0020~\textrm{h}^{-1}$ support rapid early biomass growth with sustained enzyme availability. In the hydrolysis phase, $V_{\max }=0.2179~\mathrm {g_{sugar}\,L^{-1}\,h^{-1}(U\,mL^{-1})^{-1}}$, $K_m=0.7200~\mathrm {g_{substrate}\,L^{-1}}$, and $Y_{C/B}=1.0000~\mathrm {g_{sugar}\,g_{substrate}^{-1}}$ indicate that substrate-to-sugar conversion becomes active once sufficient enzyme has accumulated. The fermentation-phase parameters allow sustained product formation without requiring unrealistically aggressive late-stage kinetics, with $\mu _{3,\max }=0.1377~\textrm{h}^{-1}$, $\mu _{d,3}=0.0050~\textrm{h}^{-1}$, $Y_{P/X}=0.3488~\mathrm {g_{product}\,g_{biomass}^{-1}}$, and $k_P=0.0045~\textrm{h}^{-1}$.

In summary, the selected solution is best viewed as a kinetically coherent endpoint-constrained grey-box reconstruction. The parameters should not be treated as uniquely identified intrinsic kinetic constants because they were selected through endpoint alignment rather than estimated from measured dynamic trajectories (von Stosch et al. 2014; Narayanan et al. 2023). Thus, the hybrid model is used here for mechanistic interpretation and hypothesis generation, not as a fully validated dynamic simulator.

**Table 8:** Best parameter set identified by endpoint-guided search at the representative operating point

Environment and surrogate target		Derived kinetic parameters	
Best endpoint model	XGB_raw	**Hybrid model 1**	
Surrogate target	2.6473	*μ *	0.2371
Temperature (${}^\circ \textrm{C}$)	50.0	$\mu _d$	0.0100
pH	5.5	$q_E$	0.0743
Pretreatment indicator	1	$k_{\textrm{deg}}$	0.0020
Initial substrate concentration ($\mathrm {g\,L^{-1}}$)	50.0	*K*	5.0000
$P_{\textrm{scale}}$	10	**Hybrid model 2**	
		$V_{\max }$	0.2179
		$K_m$	0.7200
		$Y_{C/B}$	1.0000
**Global multipliers**		**Hybrid model 3**	
$\textrm{Scale}_{\mu }$	1.1856	$\mu _{3,\max }$	0.1377
$\textrm{Scale}_{q_E}$	1.1263	$\mu _{d,3}$	0.0050
$\textrm{Scale}_{V_{\max }}$	1.0894	$Y_{P/X}$	0.3488
$\textrm{Scale}_{\mu _3}$	1.1476	$k_P$	0.0045
$\textrm{Scale}_{Y_P}$	1.1627		

The rate constants *μ *, $\mu _d$, $\mu _{3,\max }$, $\mu _{d,3}$, $k_{\textrm{deg}}$, and $k_P$ are given in $\mathrm {h^{-1}}$. The enzyme-production coefficient $q_E$ is given in $\mathrm {U\,g^{-1}\,h^{-1}}$. The carrying capacity *K* is given in $\mathrm {g\,L^{-1}}$. The hydrolysis coefficient $V_{\max }$ is given in $\mathrm {g_{sugar}\,L^{-1}\,h^{-1}(U\,mL^{-1})^{-1}}$, and $K_m$ is given in $\mathrm {g_{substrate}\,L^{-1}}$. The yield terms $Y_{C/B}$ and $Y_{P/X}$ are given in $\mathrm {g_{sugar}\,g_{substrate}^{-1}}$ and $\mathrm {g_{product}\,g_{biomass}^{-1}}$, respectively. The global multipliers, pretreatment indicator, surrogate target, and $P_{\textrm{scale}}$ are dimensionless

#### Parameter selection via endpoint-guided sweep and dynamic trajectories under accepted sets

**Fig. 8:**
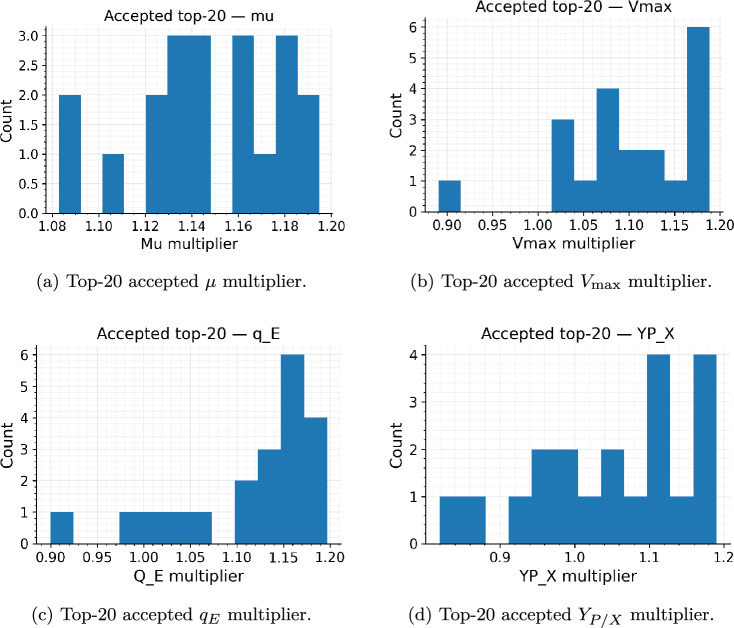
Distributions of accepted kinetic multipliers among the top-20 endpoint-aligned parameter sets identified by the XGB_raw-guided endpoint sweep

The endpoint-guided sweep produced a relatively tight top-20 set of multiplier combinations while still allowing enough flexibility to match the final ethanol target predicted by the repeated-cross-validation-selected XGB_raw endpoint surrogate. The growth multiplier in the enzyme-production block, $\textrm{Scale}_{\mu }$, was concentrated within a narrow range of approximately *1.08*–*1.20*, with most accepted values above the nominal value of unity. This pattern indicates a consistent preference for slightly faster early biomass growth than the nominal baseline, as shown in Fig. 8a. The hydrolysis-capacity multiplier, $\textrm{Scale}_{V_{\max }}$, covered a wider range of approximately *0.90*–*1.20*, but many accepted values were also above unity. This suggests that increased hydrolytic capacity was often preferred, although it was not required in every accepted solution, as shown in Fig. 8b.

The enzyme-production-rate multiplier, $\textrm{Scale}_{q_E}$, also varied within the sampled range, with several accepted values above unity. This supports the importance of early enzyme availability for endpoint agreement, as shown in Fig. 8c. The fermentation-related multipliers, $\textrm{Scale}_{\mu _3}$ and $\textrm{Scale}_{Y_P}$, showed broader accepted ranges, indicating greater equifinality in the late fermentation stage. In other words, the same endpoint prediction could be reconstructed through different combinations of downstream conversion efficiency, fermentation growth, and upstream hydrolysis, as shown in Fig. 8d.

Taken together, the accepted parameter sets suggest that endpoint agreement was achieved through moderate adjustment across process blocks rather than extreme distortion of any single kinetic term. This interpretation agrees with mechanistic CBP descriptions in which final ethanol formation emerges from the interaction of multiple stages, including enzyme accumulation, substrate deconstruction, soluble-sugar generation, and fermentation (Ahamed et al. 2021; Brethauer and Studer 2014). The phase-weighting structure preserves the intended order of enzyme production, hydrolysis, and fermentation in a dynamically plausible manner, while still allowing moderate variation in the timing of sugar release and downstream conversion across endpoint-aligned solutions. This behavior is consistent with the broader challenge of hybrid bioprocess modeling under limited observability, where endpoint data can constrain plausible dynamic behavior but cannot uniquely identify all internal kinetic contributions (von Stosch et al. 2014; Mahanty 2023).

Biological interpretation. The identified parameters are better interpreted as effective lumped CBP coefficients for the representative operating condition rather than as universal intrinsic constants. Here, the carrying-capacity term *K* provides an effective upper bound on net biomass expansion. It represents the combined influence of process limitations such as substrate accessibility, nutrient availability, inhibition, and other unmodeled constraints. Moderately elevated growth and enzyme-production terms, together with low enzyme deactivation, indicate that rapid early biomass development and sustained enzyme availability support agreement with the XGB_raw endpoint target. The hydrolysis parameters similarly indicate that sufficient upstream enzyme accumulation must be translated into effective substrate conversion before efficient downstream product formation can occur. The fermentation-related terms allow continued product formation without requiring unrealistically aggressive late-stage kinetics. Overall, the identified solution demonstrates that the ethanol endpoint is reproduced through balanced adjustment of growth, enzyme production, hydrolysis, and conversion rather than through extreme adjustment of any single process block. The distributions of these accepted kinetic multipliers are summarized in Fig. 8.

#### Phase activation and dynamic plausibility

All results in this section were obtained from *in silico* trajectories of the hybrid CBP model under the representative operating condition used for endpoint-guided calibration: $50~{}^\circ \textrm{C}$, pH 5.5, pretreated substrate, substrate concentration of $50~\mathrm {g\,L^{-1}}$, and initial state $[X_0,E_0,B_0,C_0,P_0]=[0.1,0,10,0,0]$. In the current implementation, the initial substrate state $B_0=10$ is used as a normalized reference value for dynamic simulation, while the concentration variable is used as an environmental modifier of the hydrolysis-rate coefficient. Therefore, the simulated substrate trajectory should be interpreted in relative units rather than as a direct quantitative prediction of measured substrate concentration. Biomass and enzyme trajectories are presented using the same state definitions as in the model formulation, with *X* in $\mathrm {g\,L^{-1}}$ and *E* in $\mathrm {U\,mL^{-1}}$; the substrate trajectory is shown only in normalized relative units for dynamic illustration.

The kinetic parameters were not fitted to measured time-course data. Instead, the repeated-cross-validation-selected XGB_raw endpoint surrogate provided the calibration target by evaluating a representative median-encoded input. A Monte Carlo sweep over 200 sampled multiplier sets in the range *0.8*–*1.2* then retained the top-20 parameter sets whose simulated final product best matched that endpoint (Narayanan et al. 2023; Von Stosch et al. 2021). Accordingly, the trajectories should be interpreted as endpoint-constrained dynamic reconstructions rather than experimentally validated CBP time courses.

**Fig. 9:**
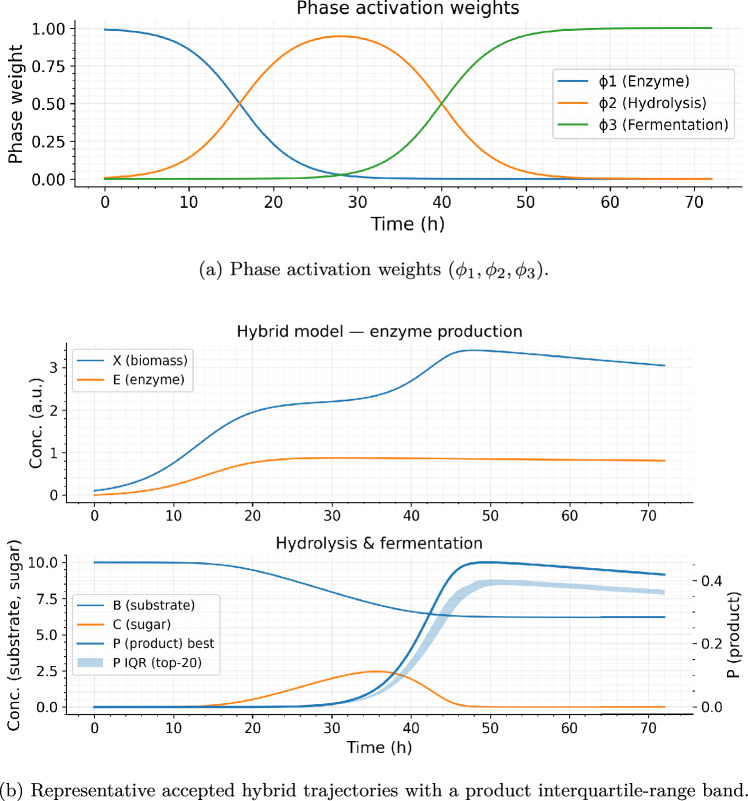
Hybrid CBP identification results based on the XGB_raw endpoint target: **a** phase-activation weights and **b** dynamically plausible trajectories under accepted parameter sets

The smooth activation functions $\phi _1$, $\phi _2$, and $\phi _3$ maintain the intended temporal order of the three CBP regimes while allowing overlap among them. The weight of enzyme production dominates at the beginning, the weight of hydrolysis peaks in the middle of the batch, and the weight of fermentation dominates in the last third, as shown in Fig. 9a. This smooth schedule prevents discontinuous switching and nonphysical behavior, such as strong product formation before appreciable hydrolysis has occurred. The phase ordering is also consistent with the general CBP concept, where enzyme production, biomass deconstruction, sugar release, and fermentation are integrated but still occur with different dominant timescales (Brethauer and Studer 2014; Ahamed et al. 2021).

The accepted trajectories are dynamically plausible and follow the expected qualitative sequence of CBP behavior. Biomass increases rapidly at the beginning and then levels off. Enzyme concentration increases early and then remains approximately stable. The insoluble substrate decreases monotonically, while soluble sugar shows a transient maximum during the middle of the run. Product formation shows a delayed sigmoidal increase. This qualitative sequence agrees with mechanistic interpretations of CBP in which upstream enzyme accumulation and substrate hydrolysis create the conditions for downstream product formation (Ahamed et al. 2021; Brethauer and Studer 2014). However, because the trajectories are constrained by endpoint agreement instead of measured time-course data, they should be viewed as plausible reconstructions, not experimentally validated dynamic profiles, as shown in Fig. 9b.

Although the best trajectory and interquartile band of accepted sets converge near the final endpoint, larger variation is observed during the transition period. This indicates that several kinetic combinations can lead to the same endpoint while differing in the timing of intermediate dynamics. This result is consistent with the equifinality expected in hybrid bioprocess models under sparse observations, where endpoint data can restrict the final product value but cannot uniquely identify all internal kinetic contributions (von Stosch et al. 2014; Mahanty 2023). The phase-weight structure and accepted trajectory family therefore support the dynamic plausibility of the grey-box formulation, while also demonstrating that endpoint agreement alone does not uniquely define the full internal trajectory.

The endpoint-guided hybrid trajectories obtained in this study should be viewed as mechanistically plausible dynamic reconstructions, not as experimentally validated optimal policies. This distinction is important when comparing the present framework with the dynamic Pareto optimization study by Yeboah et al. (2026b). That study used an *in silico* reduced-order CBP model to screen $120{,}000$ dynamic policies, from which $5{,}017$ feasible policies and 328 feasible Pareto-optimal policies were identified. The optimized dynamic policies improved ethanol titer, productivity, and conversion by *10.6~%*, *8.3~%*, and *14.3~%*, respectively, relative to the best criterion-specific static baselines. In contrast, the present study focuses on data-driven endpoint learning, endpoint-guided mechanistic reconstruction, and synthetic state-estimation feasibility. The two studies are therefore complementary: the Pareto framework addresses operating-policy selection, whereas the present grey-box framework addresses prediction, interpretation, and soft-sensing readiness when only sparse literature-derived data are available.

### State-estimation feasibility

To evaluate state-estimation performance in a controlled setting, an unscented Kalman filter (UKF) was tested entirely *in silico* using simulated measurements derived from the calibrated hybrid CBP model. In the implemented workflow, the reference trajectory was extracted from the best endpoint-aligned hybrid simulation obtained with the repeated-cross-validation-selected XGB_raw endpoint surrogate. Zero-mean Gaussian noise was added to biomass *X(t)*, soluble sugar *C(t)*, and scaled product $P(t)/P_{\textrm{scale}}$. The enzyme state was not measured directly but was reconstructed by the model-based observer. The UKF was then used to estimate the internal process states, and a simple backward smoothing pass was applied to assess whether post-processing improved the online estimates.

The results presented here should not be interpreted as experimental validation of a deployable state-estimation system. Instead, they represent a synthetic feasibility test using model-consistent measurements and a simple *post hoc* smoothing procedure. This distinction is important because the measurements were generated from the same calibrated hybrid model family used for observer propagation. Thus, the analysis tests numerical consistency and preliminary state-reconstruction feasibility under idealized conditions, not robustness to real process noise, structural plant–model mismatch, assay uncertainty, sensor drift, or missing measurements. The resulting performance metrics are summarized in Table 9.

**Fig. 10:**
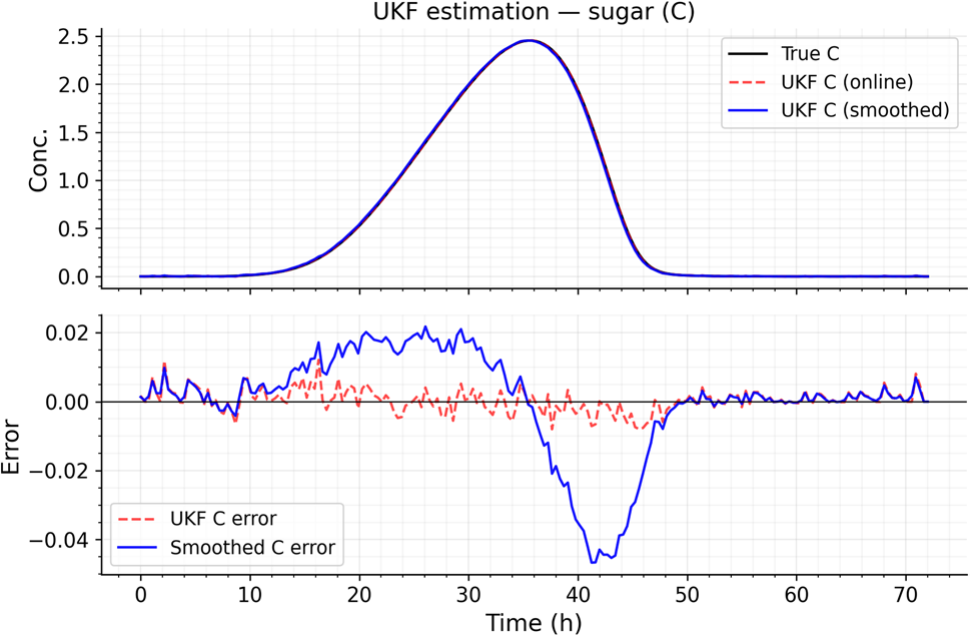
Synthetic UKF reconstruction of soluble sugar concentration *C(t)* using measurements generated from the XGB_raw-calibrated hybrid trajectory

**Fig. 11:**
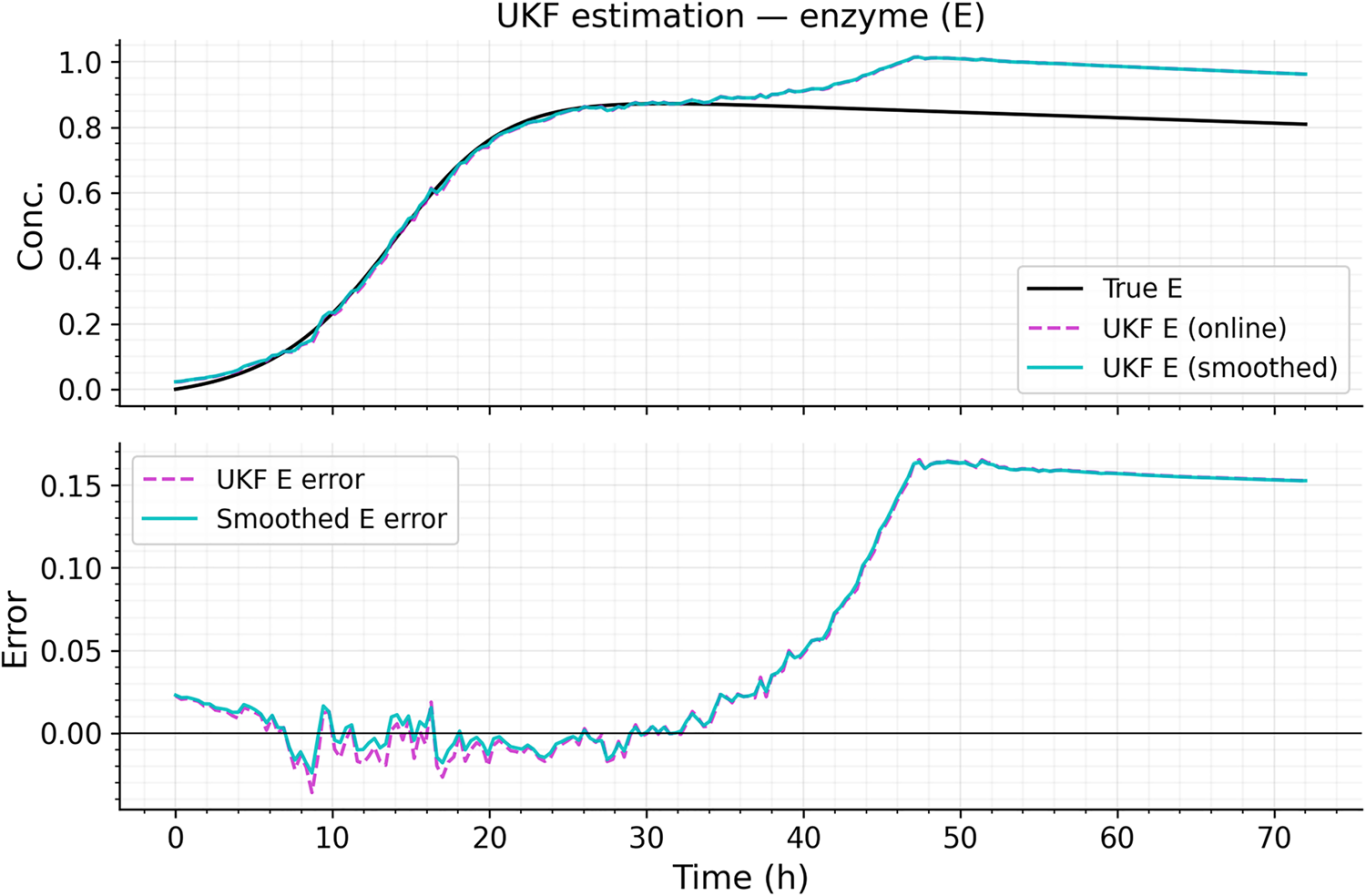
Synthetic UKF reconstruction of enzyme concentration *E(t)* using measurements generated from the XGB_raw-calibrated hybrid trajectory

**Fig. 12:**
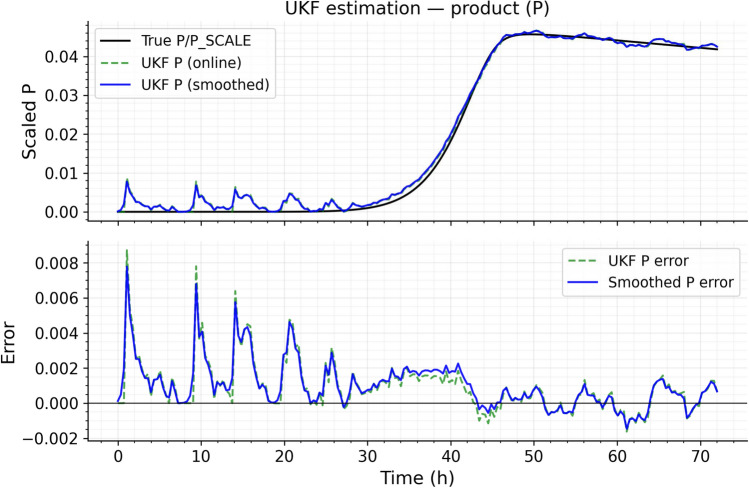
Synthetic UKF reconstruction of scaled product concentration $P(t)/P_{\textrm{scale}}$ using measurements generated from the XGB_raw-calibrated hybrid trajectory

The hybrid observer recovers the main states with generally high fidelity, although performance varies across variables. The online filter reconstructs sugar with very low absolute error, whereas the smoothed estimate introduces larger transient errors around the turnover region, as shown in Fig. 10. The enzyme state is reasonably well captured in timing and overall trend, but both online and smoothed estimates show a persistent late-stage positive bias, as shown in Fig. 11. The scaled-product state is also well reconstructed once accumulation begins, with small absolute errors at early times when the signal is close to zero, as shown in Fig. 12.

These trends are consistent with the broader bioprocess soft-sensing literature, where directly measured or strongly constrained states are generally easier to estimate than latent biological states that are only indirectly informed by the measurement set (Rathore et al. 2021; Alexander et al. 2020; Lyubenova et al. 2021). They are also consistent with recent CBP digital-twin work showing that sensor-set selection strongly affects state observability, parameter identifiability, and UKF-based soft-sensing performance (Yeboah et al. 2026c). In the present synthetic test, sugar and scaled product are measured and therefore remain strongly constrained, whereas enzyme activity is reconstructed indirectly through the model structure. The enzyme bias therefore highlights an important limitation for future CBP monitoring: enzyme activity may require direct assay information, an additional proxy measurement, or a stronger dynamic model before it can be estimated reliably in experimental operation.

Because MRPE and MRPU are normalized by the magnitude of the predicted signal, their values may increase when predicted concentrations are close to zero. For states that approach zero during part of the trajectory, such as sugar and scaled product at early times, small absolute errors can translate into large relative percentages. Therefore, the low RMSE and high $R^2$ values are more informative measures of absolute reconstruction quality in this synthetic setting. Overall, the UKF accurately reconstructs the sugar and scaled-product states and captures the enzyme dynamics with useful but lower fidelity. However, smoothing does not consistently improve the estimates and should therefore be applied selectively rather than as a default post-processing step.

These results support the use of the XGB_raw-calibrated phase-structured hybrid model as a candidate soft-sensing scaffold for internal CBP states. However, this support remains at the numerical feasibility level. Before practical deployment, the estimator must be tested under real assay noise, sensor drift, missing measurements, disturbances, and structural plant–model mismatch. Formal observability and identifiability analyses should also be performed for the selected measurement configuration because recursive state estimation cannot recover unobservable or weakly informed states regardless of the estimator used (Golabgir et al. 2015; Alexander et al. 2020; Yeboah et al. 2026c). The next main step is therefore experimental validation using time-resolved CBP measurements of biomass, enzyme activity, residual solids, soluble sugars, and ethanol.

**Table 9:** Performance of the unscented Kalman filter on the best simulated trajectory for online and smoothed estimates. The results are based on synthetic model-generated measurements from the XGB_raw-calibrated hybrid simulator and therefore indicate numerical feasibility rather than experimental validation

Signal	RMSE	MRPE (%)	MRPU (%)	$\boldsymbol{R^2}$
Enzyme (E)	0.0993	11.35	26.98	0.8737
Sugar (C)	0.0033	30.05	89.71	1.0000
Product (*P*, scaled)	0.0018	31.51	49.12	0.9925
Enzyme (E), smoothed	0.0992	11.25	25.84	0.8738
Sugar (C), smoothed	0.0151	29.21	398.40	0.9997
Product (*P*, scaled), smoothed	0.0017	34.58	44.97	0.9930

### Comparative appraisal and practical implications

The present framework extends endpoint-focused CBP modeling by using the learned ethanol endpoint not only for prediction but also as a calibration signal for mechanistically interpretable dynamic reconstruction and synthetic state-estimation analysis. This distinguishes the present work from purely endpoint-level literature-derived CBP models, in which prediction is not directly linked to dynamic trajectory reconstruction (Yeboah et al. 2026a).

The endpoint-learning results show that boosting-based tree ensembles were the most robust model family for the curated dataset. Repeated cross-validation selected XGB_raw, whereas the independent hold-out subset favored HGB_log and HGB_raw. This criterion sensitivity indicates that the leading models were closely matched and is best interpreted at the model-family level rather than for a single universally superior surrogate. This result also differs from earlier CBP machine-learning work, where Gaussian-process regression performed best on a much smaller dataset (Yeboah et al. 2024). The difference is likely due to the larger sample size, broader feature representation, and greater heterogeneity of the present 540-run, 90-feature dataset.

The feature-attribution results are consistent with the experimental CBP literature: hemicellulose content, substrate concentration, temperature, residence time, enzyme-assisted pretreatment, pH, cellulose content, and mixing rate emerged as important predictors. These variables reflect the combined effects of substrate accessibility, hydrolysis efficiency, microbial capability, and fermentation conditions rather than a single dominant control variable (Brethauer and Studer 2014; Kavitha et al. 2022). The attributions should therefore be interpreted as model-based associations within the compiled literature corpus, not as causal effects.

The main practical value of the framework is that it links a fast endpoint surrogate to a phase-structured simulator. The endpoint model can screen operating and biological descriptors, while the grey-box layer provides a mechanistic scaffold for interpreting whether endpoint performance is associated with early growth, enzyme formation, hydrolysis, sugar accumulation, or downstream conversion. The synthetic unscented Kalman filter study further suggests that the same structure can support preliminary soft-sensing analysis, especially for sugar and scaled product, although enzyme activity remains more difficult because it is only indirectly constrained (Narayanan et al. 2020; Gallego et al. 2020; Alexander et al. 2020). This interpretation is consistent with recent CBP digital-twin work showing that sensor-set selection strongly affects state observability, parameter identifiability, and UKF-based reconstruction performance (Yeboah et al. 2026c).

Overall, the contribution is the integration of repeated-cross-validation-based endpoint model selection, feature attribution, endpoint-guided hybrid reconstruction, and preliminary unscented Kalman filter feasibility analysis into a single workflow for sparse, literature-derived CBP data. The framework should be regarded as a hypothesis-generation and process-interpretation tool until external endpoint validation, time-resolved experimental measurements, and sensor-set-specific observability assessment are available.

### Limitations and future work

The main limitation is the absence of experimentally measured time-resolved CBP trajectories. The simulated profiles are therefore endpoint-constrained dynamic reconstructions, not validated kinetic trajectories (Foster et al. 2022; Albino et al. 2024). Future work should validate the phase-structured simulator using synchronized measurements of biomass, enzyme activity, residual substrate, soluble sugars, and ethanol.

A second limitation is the lack of an external endpoint-validation dataset beyond the compiled 540-run literature corpus. The hold-out subset provides an internal test within the curated dataset, but not independent experimental validation. Future studies should test the endpoint surrogate on new CBP campaigns covering different feedstocks, organisms, pretreatments, and operating regimes.

A third limitation is the heterogeneity of the literature-derived data. The endpoint model should be viewed as an empirical summarizer of the assembled corpus rather than as a universal CBP predictor (Fisher et al. 2022; Huntington et al. 2023; Jurinjak Tušek et al. 2024). Residual screening improved internal consistency, but the removed records should not be assumed to be experimentally invalid. Future work should also examine reduced-feature models or feature-distillation strategies to improve parsimony.

Model selection also remains uncertain because repeated cross-validation and hold-out evaluation favored different boosting models. This behavior is expected when models have similar performance and validation estimates have non-negligible variance (Kohavi 1995; Cawley and Talbot 2010; Varma and Simon 2006; Varoquaux 2018). External validation or nested repeated validation on expanded datasets will be needed to determine whether XGB_raw, HGB_log, or another boosting-based model generalizes best.

The biological model is deliberately lumped. Endpoint-guided calibration identifies parameter sets consistent with the surrogate endpoint, but endpoint data alone cannot uniquely resolve the internal contributions of growth, enzyme production, hydrolysis, and fermentation. Future work should include identifiability analysis, Bayesian or multi-start parameter estimation, and explicit oxygen dynamics when suitable measurements become available (Mahanty 2023; Wang et al. 2023; Raue et al. 2009).

Finally, the unscented Kalman filter study is a synthetic feasibility test rather than experimental soft-sensor validation. Real deployment would require testing under assay noise, sensor drift, missing measurements, disturbances, and plant–model mismatch. Formal observability and identifiability analyses should also be performed for candidate measurement configurations because recursive state estimation cannot recover unobservable or weakly informed states regardless of the estimator used (Golabgir et al. 2015; Alexander et al. 2020; Yeboah et al. 2026c). Future extensions should connect the endpoint-guided grey-box model with time-resolved validation, sensor-set design, dynamic optimization, model predictive control, and uncertainty-aware operating-policy selection.

## Summary and conclusions

This study presents an endpoint-guided grey-box framework for consolidated bioprocessing that combines data-driven endpoint prediction, phase-structured mechanistic reconstruction, and synthetic state-estimation analysis. Several nonlinear regressors were evaluated using both log-transformed and raw endpoint targets. Repeated cross-validation selected XGB_raw as the final endpoint surrogate, with mean repeated-cross-validation RMSE *=1.566 ± 0.465*. The hold-out subset favored HGB_log and HGB_raw, indicating that the leading boosting-based models were closely matched and that exact ranking depended on the evaluation criterion.Thus, the results indicate that boosting-based tree ensembles provide reliable endpoint prediction for the curated CBP dataset, while the exact ranking of individual models should not be interpreted as evidence of universal superiority.

Out-of-fold residual screening improved the internal consistency of the literature-derived dataset. The OOF RMSE decreased from 6.450 to 1.819, while OOF $R^2$ increased from 0.799 to 0.961. For the selected XGB_raw model, hold-out performance improved from RMSE *=7.491*, MAE *=2.798*, and $R^2=0.821$ before trimming to RMSE *=2.025*, MAE *=1.253*, and $R^2=0.940$ after trimming. These results demonstrate strong internal predictive performance within the compiled corpus, although external endpoint validation is still required.

The selected endpoint surrogate was coupled to a three-phase simulator representing enzyme production, hydrolysis, and fermentation. Endpoint-guided calibration identified plausible parameterizations in which the target endpoint was reproduced through moderate changes in growth, enzyme production, hydrolytic capacity, and product formation. Feature attribution indicated that endpoint variability was mainly associated with hemicellulose content, substrate concentration, temperature, residence time, enzyme-assisted pretreatment, pH, cellulose content, and mixing rate. The synthetic unscented Kalman filter study further showed that the calibrated hybrid structure can support preliminary reconstruction of sugar and scaled-product states, while enzyme dynamics remained more difficult to estimate.

Overall, the framework provides a pragmatic grey-box strategy for CBP under sparse endpoint-data conditions. It links repeated-cross-validation-based endpoint model selection with mechanistically interpretable endpoint-constrained reconstruction and preliminary soft-sensing analysis. Future work should focus on external endpoint validation, time-resolved experimental validation, explicit oxygen modeling where data permit, and integration with process monitoring, optimization, and control.

## Supplementary Materials

A summary of the literature-derived CBP ethanol dataset, including the biomass/reference grouping, is provided in Table S1, and the compact catalog of encoded endpoint-learning features and the target variable is provided in Table S2. Additional residual-screening diagnostics are included in Tables S3 and S4. The distributions of the highest-shift features between retained and deleted samples after out-of-fold residual screening are summarized in Table S3. The permutation feature-importance results before trimming, together with the corresponding post-trimming values, are reported in Table S4. Supplementary SHAP dependence plots for hemicellulose content, pH, mixing rate, and residence time are provided in Supplementary Figures 1–4. The 540-run endpoint dataset was compiled from the following literature sources: (Althuri et al. 2017; Argyros et al. 2011; Anandharaj et al. 2020; Brethauer and Studer 2014; Bu et al. 2019; Chung et al. 2015; Davison et al. 2019; Dempfle 2022; Drosos et al. 2021; Fan et al. 2012; Gupte et al. 2024; He et al. 2011; Hong et al. 2014; Jeon et al. 2009; Jiang et al. 2020; Jin et al. 2012; Kavitha et al. 2021, 2022, 2023; Liu et al. 2016; Maleki et al. 2021; Malherbe et al. 2023; Mattila et al. 2018; Minnaar and den Haan 2023; Mohapatra et al. 2020; Munoz-Gutierrez et al. 2012; Nakatani et al. 2013; Nongthombam et al. 2022; Pang et al. 2018; Papathoti et al. 2024; Park et al. 2012; Perez et al. 2023; Ramos et al. 2023; Restiawaty et al. 2023; Ryu et al. 2011; Vkdo et al. 2025; Selvakumar et al. 2019; Singh et al. 2020; Sukma et al. 2026; Sun et al. 2012; Svetlitchnyi et al. 2013; Tang et al. 2018; Tsai et al. 2009, 2010, 2013; Vaid et al. 2017, 2021; Wang et al. 2022, 2024; Wen et al. 2010; Xiong et al. 2018; Zhang et al. 2023; Zuroff et al. 2013; Xu and Tschirner 2011).

## Supplementary Information

Supplementary Material1

## Data Availability

The original contributions presented in this study are included in the article and Supplementary Material. Further inquiries can be directed to the corresponding author.
