# Correction: Grey-box modeling framework for consolidated bioprocessing systems: an endpoint-guided approach

**DOI:** 10.1186/s40643-026-01108-2

**Published:** 2026-08-17

**Authors:** Mark Korang Yeboah, Dirk Söffker

**Affiliations:** Chair of Dynamics and Control, University of Duisburg–Essen, Lotharstraße, 47057 Duisburg, NRW Germany

**Correction to: Bioresources and Bioprocessing (2026) 13:103** 10.1186/s40643-026-01101-9

In the article (Yeboah and Söffker 2026) it has come to the author’s attention that the graphical abstract was inadvertently omitted in the article.

Now the graphical abstract has been included in the article.

Graphical Abstract:

**Figure:**
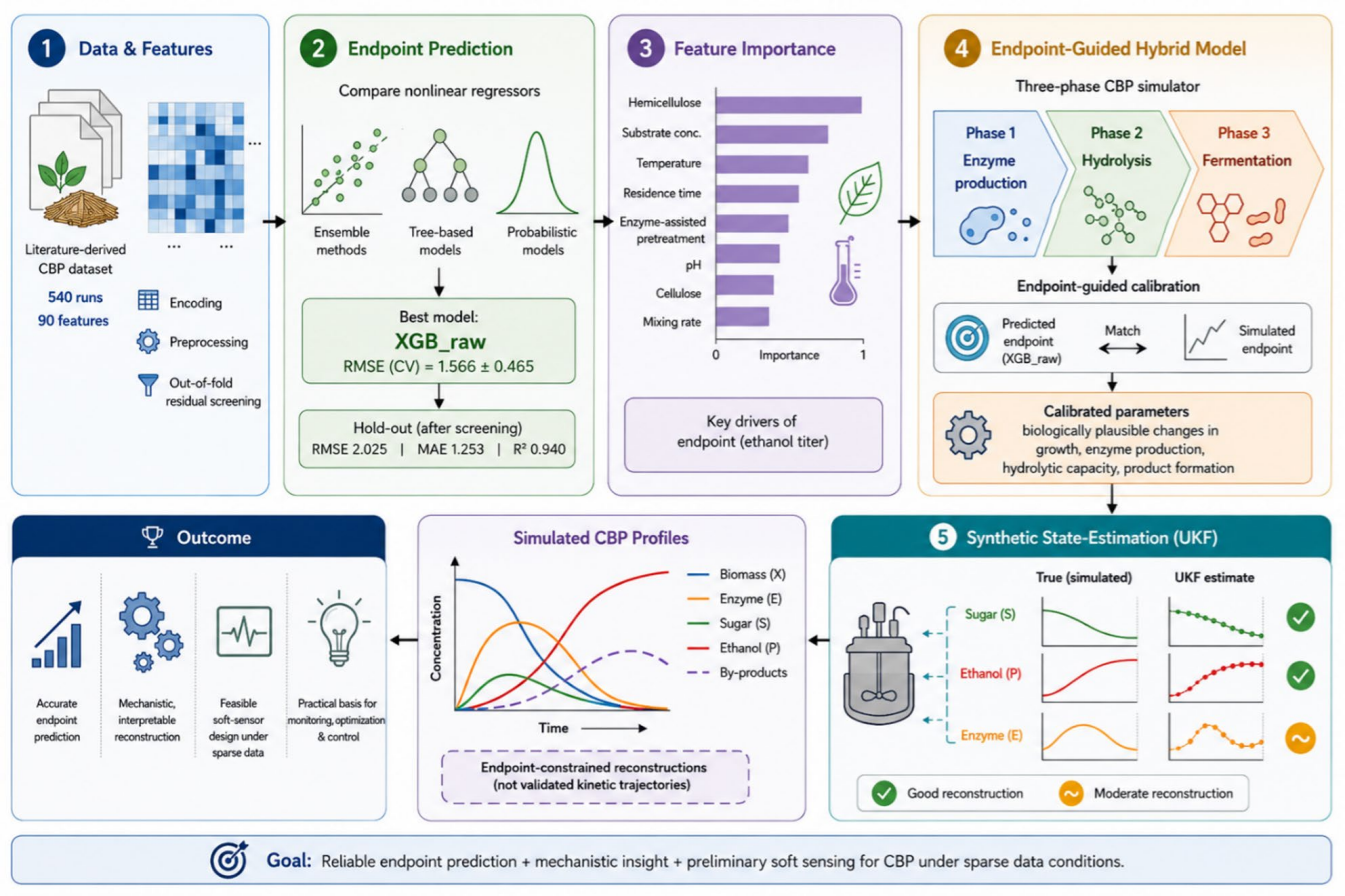

The original article has been corrected.

## References

[CR1] Yeboah MK, Söffker D (2026) Grey-box modeling framework for consolidated bioprocessing systems: an endpoint-guided approach. Bioresour Bioprocess 13:103 42472721 10.1186/s40643-026-01101-9 PMC13381484

