# Supplementary material for: Grey-box modeling framework for consolidated bioprocessing systems: an endpoint-guided approach
Source: Bioresour Bioprocess. 2026 Jul 20;13(1):103. doi: 10.1186/s40643-026-01101-9 (PMC13381484; doi:10.1186/s40643-026-01101-9)
Supplement: Supplementary file 1 — Supplementary Material1 [file 40643_2026_1101_MOESM1_ESM.pdf]

Supplementary Material

Table S1: Summary of the literature-derived ethanol dataset used for CBP modeling ($n = 540$), aggregated by biomass/reference.

Biomass	Number of data points	Reference
Mixed lignocellulosics	30	Althuri (2017)
Avicel	3	Argyros (2011)
Avicel	1	Anandharaj (2020)
Avicel; Wheat straw	3	Brethauer (2014)
PASC[a]	1	Anandharaj (2020)
PASC[a]	1	Liu (2016)
PASC[a]	1	Nakatani (2013)
PASC[a]	1	Ryu & Karim (2011)
PASC[a]	1	Tang (2018)
PASC[a]	1	Tsai (2009)
PASC[a]	1	Tsai (2010)
PASC[a]	1	Tsai (2013)
PASC[a]	1	Wen (2010)
Cellulose	1	Bu (2019)
Cellulose	3	Dempfle (2022)
Cellulose	1	Zuroff (2013)
Avicel; Switchgrass; Cellobiose	9	Chung (2015)
Corn cob	1	Davison (2019)
Starch	10	Drosos (2021)
Avicel	1	Fan (2012)
Bread and pasta waste; Discolored rice; OFMSW[b] blend; OFMSW[b]	10	Gupte (2024)
Solka Floc	5	He (2011)
Corn cob	1	Hong (2014)
β-Glucan	1	Jeon (2009)
Corn cob; Xylan	2	Jiang (2020)
Corn stover	3	Jin (2012)
A. ascalonicum leaves	114	Kavitha (2022)
Sargassum wightii	31	Kavitha (2023)
Nannochloropsis gaditana	4	Kavitha S. (2021)
Potato waste	5	Maleki (2021)
Sorghum bran; Sorghum flour; Triticale bran; Triticale flour	8	Malherbe (2023)
Barley straw; Core board; Core board + spruce sawdust; Wheat straw + barley mash	5	Mattila (2018)

Continued on next page

Biomass	Number of data points	Reference
Avicel	5	Minnaar & den Haan (2023)
Denannath grass; Glucose; Glucose + xylose; Xylose	45	Mohapatra (2020)
Cellobiose	1	Munoz-Gutierrez (2012)
Ficus fruits	2	Nongthombam (2022)
Microcrystalline cellulose; Salix psammophila	16	Pang (2018)
Cassava stem; Cellobiose + xylose	3	Papathoti NK (2024)
Solka Floc	8	Park (2012)
Sugarcane bagasse; Synthetic medium	2	Perez CL (2023)
Synthetic medium	5	Ramos (2023)
Vetiver leaves	1	Restiawaty (2023)
Cassava stem; Corn cob; Corn stover; Hemp fiber; Hemp hurds; Paper sludge; Hemp cellulose; Rice straw; Sugarcane bagasse; Switchgrass	18	Schmidt (2025)
Cassava stem	31	Selvakumar (2019)
Rice straw	1	Singh (2020)
Rice straw	20	Sukma ACT (2026)
Arabinoxylan	1	Sun (2012)
Avicel; Glucose; Poplar; Miscanthus	23	Svetlitchnyi (2013)
Pine needles	1	Vaid (2017)
Saccharum spontaneum	1	Vaid (2021)
Avicel + xylan	3	Wang (2022)
Sweet potato residue	12	Wang (2024)
Cellulose + hemicellulose sugars	3	Xiong (2018)
Cellobiose; Glucose; Hemicellulose; Microcrystalline cellulose; Xylose	65	Xu (2011)
Avicel; Corn cob; Xylan; Xylose	12	Zhang (2023)

[a] PASC = phosphoric acid-swollen cellulose; [b] OFMSW = organic fraction of municipal solid waste.

Table S2: Compact catalog of encoded endpoint-learning features and target variable from the dataset.

Feature	Feature group	Type	Observed range	Short description
Temperature (°C)	Operating conditions	Continuous	25–75	Temperature
pH		Continuous	3–9	pH
residence time (hr)		Continuous	6–3504	Residence time
Cellulose (%)	Substrate / composition	Continuous	1.7–90	Cellulose fraction
Hemicellulose (%)		Continuous	0.3–35.24	Hemicellulose fraction
Initial ethanol added (g/L)		Continuous	0–4	Initial ethanol concentration
Lignin (%)		Continuous	0.4–37.5	Lignin fraction
Substrate concentration (g/L)		Continuous	2–367	Substrate concentration
feedstock_class_ lignocellulosic		Binary	0–1	Lignocellulosic feedstock
feedstock_class_ model_carbohydrate				Model carbohydrate feedstock
feedstock_class_other				Other feedstock class
pretreatment_class_ acid	Pretreatment	Binary	0–1	Acid pretreatment class
pretreatment_class_ alkali				Alkali pretreatment class
pretreatment_class_ biological				Biological pretreatment class
pretreatment_class_ enzyme_assisted				Enzyme-assisted pretreatment class
pretreatment_class_ none				No pretreatment class
pretreatment_class_ other				Other pretreatment class
pretreatment_class_ oxidative				Oxidative pretreatment class
pt_conc_unit_v/v		Binary	0–1	Pretreatment concentration unit v/v

Continued on next page

Feature	Feature group	Type	Observed range	Short description
pt_conc_unit_w/v				Pretreatment concentration unit w/v
pt_conc_unit_w/w				Pretreatment concentration unit w/w
pt_conc_unit_wt%				Pretreatment concentration unit wt%
pt_has_ionic_liquid		Binary	0–1	Ionic liquid used in pretreatment
pt_has_organosolv				Organosolv used in pretreatment
pt_has_oxidant				Oxidant used in pretreatment
pt_has_steam_explosion				Steam explosion used in pretreatment
pt_has_ultrasound				Ultrasound used in pretreatment
pt_logR0		Continuous	1.833–3.158	Severity index
pt_method_family_acid		Binary	0–1	Acid pretreatment method
pt_method_family_alkali				Alkali pretreatment method
pt_method_family_hydrothermal				Hydrothermal pretreatment method
pt_method_family_ionic_liquid_DES				Ionic liquid / DES pretreatment method
pt_method_family_none				No pretreatment method
pt_method_family_other				Other pretreatment method
pt_method_family_biological				Biological pretreatment method
pt_method_family_organosolv				Organosolv pretreatment method
pt_method_family_steam_explosion				Steam-explosion pretreatment method
pt_method_family_oxidative				Oxidative pretreatment method

Continued on next page

Feature	Feature group	Type	Observed range	Short description
pt__neutralized		Binary	0–1	Pretreatment neutralized
pt__parse__confidence__score		Discrete	1–2	Pretreatment parse confidence
pt__primary__reagent__H2SO4		Binary	0–1	H_2SO_4 primary reagent
pt__primary__reagent__HNO3				HNO_3 primary reagent
pt__primary__reagent__IL				Ionic liquid primary reagent
pt__primary__reagent__NH3				NH_3 primary reagent
pt__primary__reagent__NaOH				NaOH primary reagent
pt__primary__reagent__water				Water primary reagent
pt__primary__reagent__H2O2				H_2O_2 primary reagent
pt__reagent__conc		Continuous	1–15	Reagent concentration
pt__temperature__C		Discrete	30–140	Pretreatment temperature (°C)
pt__time__min		Continuous	15–30240	Pretreatment time (min)
pt__solids__pct		Continuous	7.5–7.5	Pretreatment solids content (%)
pt__pH		Continuous	5.0–11.5	Pretreatment pH
pt__washed		Binary	0–1	Pretreated substrate washed
genus__count__total	Culture / organism	Discrete	1–3	Total number of genera
inoculum__fraction__vv		Continuous	0.01–0.2	Inoculum fraction (v/v)
kingdom__mix__bacteria__only		Binary	0–1	Bacteria-only consortium
kingdom__mix__fungi+yeast				Fungi + yeast consortium
kingdom__mix__fungi__only				Fungi-only consortium

Continued on next page

Feature	Feature group	Type	Observed range	Short description
kingdom_mix_mixed_other				Other mixed consortium
kingdom_mix_yeast_only				Yeast-only consortium
microbe_can_ferment_cellulose_directly		Binary	0–1	Direct cellulose fermentation capability
microbe_can_use_cellobiose				Cellobiose utilization capability
microbe_can_use_starch_inulin				Starch/inulin utilization capability
microbe_can_use_xylose				Xylose utilization capability
microbe_has_bacteria		Binary	0–1	Bacteria present
microbe_has_cellulolytic_hydrolyzer				Cellulolytic hydrolyzer present
microbe_has_dedicated_fermenter				Dedicated fermenter present
microbe_has_dedicated_hydrolyzer				Dedicated hydrolyzer present
microbe_has_engineered_strain				Engineered strain present
microbe_has_fungus				Fungus present
microbe_has_strict_anaerobe				Strict anaerobe present
microbe_has_yeast				Yeast present
microbe_is_thermophilic				Thermophilic microorganism present
microbe_trait_confidence_score		Discrete	1–2	Microbial trait parse confidence
system_type_co_culture		Binary	0–1	Co-culture system
system_type_mono				Monoculture system
Mixing rate (rpm)	Reactor / mixing	Continuous	0–400	Mixing rate
Working volume (L)		Continuous	0.004–4	Working volume
mixing_mode_shaker		Binary	0–1	Shaker mixing mode
mixing_mode_static				Static mixing mode
mixing_mode_stirred				Stirred mixing mode

Continued on next page

Feature	Feature group	Type	Observed range	Short description
mixing_mode_ unknown				Unknown mixing mode
reactor_scale_ bioreactor		Binary	0–1	Bioreactor scale
reactor_scale_flask				Flask scale
Cellulose (%)_missing	Missing-value indicators	Binary	0–1	Missing value for Cellulose (%)
Lignin (%)_missing				Missing value for Lignin (%)
Mixing rate (rpm)_ missing				Missing value for Mixing rate (rpm)
Substrate concentration (g/L)_missing				Missing value for Substrate concentration (g/L)
Temperature (°C)_ missing				Missing value for Temperature (°C)
Working volume (L)_ missing				Missing value for Working volume (L)
inoculum_fraction_ vv_missing				Missing value for inoculum fraction (v/v)
pH_missing				Missing value for pH
pt_conc_unit_nan				Missing value for pretreatment concentration unit
pt_detox_method_nan				Missing value for pretreatment detoxification method
pt_primary_reagent_ nan				Missing value for pretreatment primary reagent
residence time (hr)_ missing				Missing value for residence time (hr)
ethanol_is_estimated	Data quality / encoding	Binary	0–1	Ethanol value estimated
ethanol_titer_gL_std	Target / output	Continuous	standardized	Standardized endpoint ethanol titer

Table S2 provides a compact feature catalog for the encoded endpoint-learning variables and target variable from the dataset. Within contiguous indicator-variable blocks, blank cells in

the *Type* and *Observed range* columns inherit the same binary 0/1 coding as the first row of the block, where 1 denotes yes/presence and 0 denotes no/absence.

Additional residual-screening diagnostics

The following tables provide tabular diagnostics for the observations deleted by the out-of-fold residual-screening step. The distributions of the highest-shift features between retained and deleted samples are compared in the supplementary material, Table S3. Permutation importance before trimming is also reported and compared with the corresponding post-trimming importance values in the supplementary material, Table S4.

Table S3: Key feature-distribution differences between retained and deleted samples after out-of-fold residual screening. Values are reported as mean (median).

**Feature**	**Retained**	**Deleted**	**SMD**	**KS** p
Temperature (°C)	46.001 (37.000)	35.081 (34.000)	-1.017	9.0×10^{-15}
Substrate concentration (g/L)	29.151 (10.000)	101.194 (50.000)	0.925	3.5×10^{-27}
Bacteria-only system	0.844 (1.000)	0.452 (0.000)	-0.899	2.1×10^{-14}
pH	7.345 (7.200)	6.353 (7.200)	-0.821	7.7×10^{-9}
Yeast-only system	0.086 (0.000)	0.415 (0.000)	0.816	4.0×10^{-10}
Other feedstock class	0.126 (0.000)	0.459 (0.000)	0.785	2.0×10^{-10}

Notes: SMD = standardized mean difference, calculated as deleted minus retained; KS p = p-value from the two-sample Kolmogorov–Smirnov test. For binary variables, means indicate the fraction of samples with value 1.

Table S4: Permutation feature importance before and after trimming for the highest-ranked non-missingness features in the untrimmed hold-out analysis. Values are reported as mean decrease in R^2 after permutation, ΔR^2, with standard deviation over repeats.

**Feature**	**Before**	**After**	**Change**
Substrate concentration (g/L)	0.438 ± 0.045	0.254 ± 0.014	-0.183
Bacteria-only system	0.243 ± 0.027	0.004 ± 0.001	-0.239
Yeast-only system	0.085 ± 0.009	0.017 ± 0.001	-0.068
Bacteria-containing microbe	0.076 ± 0.009	0.002 ± 0.001	-0.074
Other feedstock class	0.075 ± 0.009	0.022 ± 0.001	-0.053
Co-culture system	0.054 ± 0.012	0.050 ± 0.041	-0.003
Temperature (°C)	0.053 ± 0.015	0.193 ± 0.046	0.140
Monoculture system	0.037 ± 0.009	-0.000 ± 0.000	-0.038
Lignocellulosic feedstock	0.028 ± 0.008	0.003 ± 0.003	-0.025
Total genus count	0.020 ± 0.007	-0.000 ± 0.001	-0.021
No pretreatment	0.019 ± 0.004	-0.000 ± 0.000	-0.019
Cellulolytic hydrolyzer present	0.012 ± 0.003	0.001 ± 0.001	-0.012

Notes: Before = permutation importance from the untrimmed hold-out analysis; After = corresponding permutation importance after residual-based trimming; Change = After minus Before. Importance is reported as mean $\Delta R^2 \pm$ standard deviation over permutation repeats. Negative values close to zero indicate negligible or unstable permutation importance.

Supplementary SHAP dependence plots

Additional SHAP dependence plots for hemicellulose content, pH, mixing rate, and residence time are shown in Figures 1–4.

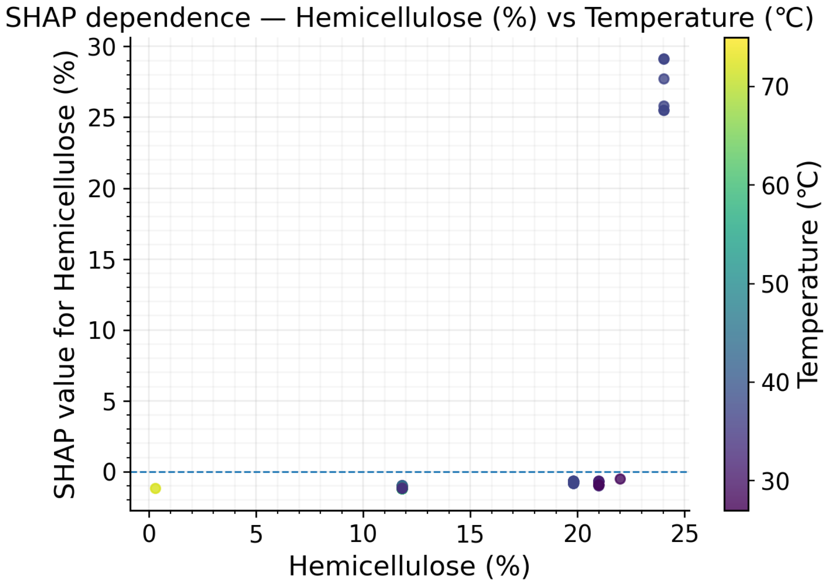

Figure 1: Supplementary SHAP dependence plot for hemicellulose in the final `XGB_raw` endpoint model. Point color indicates temperature, which was used as the interaction variable. Positive SHAP values indicate an increase in the predicted endpoint ethanol titer, whereas negative SHAP values indicate a decrease.

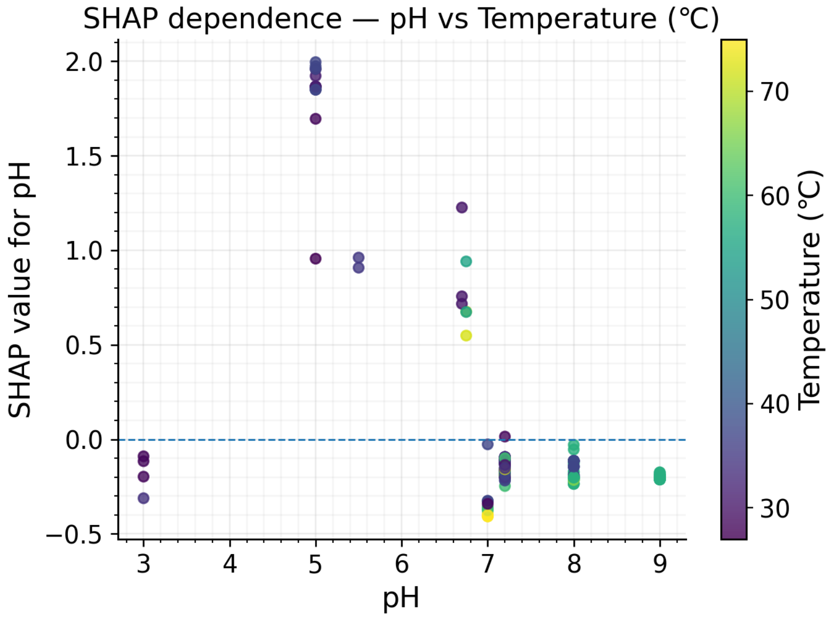

Figure 2: Supplementary SHAP dependence plot for pH in the final `XGB_raw` endpoint model. Point color indicates temperature, which was used as the interaction variable. Positive SHAP values indicate an increase in the predicted endpoint ethanol titer, whereas negative SHAP values indicate a decrease.

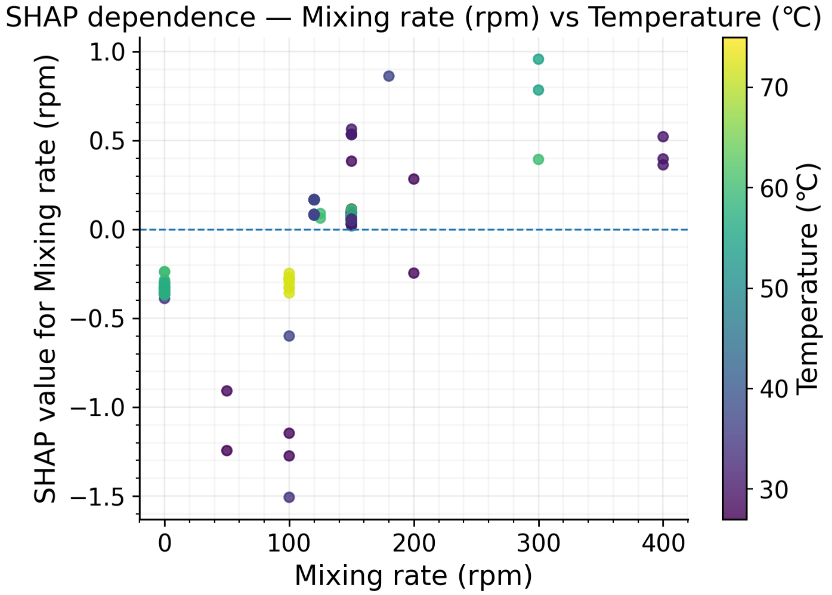

Figure 3: Supplementary SHAP dependence plot for mixing rate in the final `XGB_raw` endpoint model. Point color indicates temperature, which was used as the interaction variable. Positive SHAP values indicate an increase in the predicted endpoint ethanol titer, whereas negative SHAP values indicate a decrease.

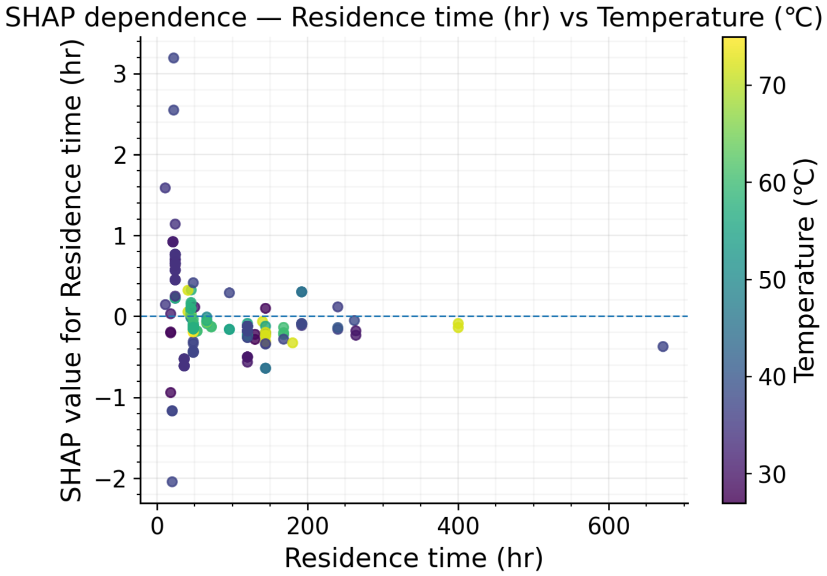

Figure 4: Supplementary SHAP dependence plot for residence time in the final `XGB_raw` endpoint model. Point color indicates temperature, which was used as the interaction variable. Positive SHAP values indicate an increase in the predicted endpoint ethanol titer, whereas negative SHAP values indicate a decrease.
